# Temporal Transcriptional Regulation of Human Neuronal Differentiation via Forward Programming

**DOI:** 10.1002/advs.202510641

**Published:** 2025-11-23

**Authors:** Lingling Zhu, Weiguang Wang, Jian Zhang, Lei Liu, Minpeng Huang, Lei Diao, Shuang Feng, Qiong Yang, Hao Qiu, Bing Pan, Renee A Reijo Pera, Ji Liu, Ninuo Xia, Fang Fang

**Affiliations:** ^1^ Center for Reproduction and Genetics Department of Obstetrics and Gynecology The First Affiliated Hospital of USTC Center for Advanced Interdisciplinary Science and Biomedicine of IHM Division of Life Sciences and Medicine University of Science and Technology of China Hefei 230001 China; ^2^ Medical School Fuyang Normal University Fuyang Anhui 230031 China; ^3^ CodeR Therapeutics, Ltd. Hefei Anhui 230027 China; ^4^ McLaughlin Research Institute Great Falls MT 59405 USA; ^5^ Faculty of Life and Health Sciences Shenzhen University of Advanced Technology Shenzhen Guangdong 518000 China

**Keywords:** developmental timing, gene regulatory networks (GRNs), multi‐omic analysis, neuronal differentiation, stepwise cellular differentiation, time‐course single‐cell RNA sequencing, transcription factor‐induced forward programming

## Abstract

Human pluripotent stem cells (hPSCs) serve as a powerful model for studying human neuronal differentiation, yet the temporal control of this process remains poorly understood. This study compares two differentiation systems with distinct timing of differentiation: transcription factor (TF)‐induced forward programming and stepwise cellular differentiation by dual‐SMAD (DS) inhibition. The analyses reveal that divergent cellular trajectories drive distinct neurogenesis timing. Multi‐omic analysis identifies crucial gene regulatory networks (GRNs) that govern cell fate determination and timing control. Perturbation of these GRNs modulates the timing of neurogenesis and neuronal maturation. Specifically, OLIG family TFs, enriched in the TF‐induced system, promoted cell cycle exit via NOTCH signaling regulation; their ablation delays neurogenesis in this system. Additionally, NEUROD2 overexpression after neurogenesis accelerated in vitro neuronal maturation in both TF‐ and DS‐induced differentiating cells by enhanced activation of maturation gene modules. These findings elucidate transcriptional mechanisms governing differentiation timing and provide a framework for rationally designing timing‐controlled in vitro differentiation strategies.

## Graphical abstract

Single‐cell profiling of TF‐induced forward programming versus stepwise dual‐SMAD differentiation reveals that divergent trajectories set the pace of neurogenesis. OLIG TFs advance cell‐cycle exit via NOTCH modulation, while NEUROD2 later accelerates maturation. The study elucidates transcriptional mechanisms governing differentiation timing, providing a reference for rationally designing timing‐controlled in vitro differentiation strategies.

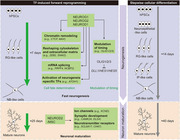

## 1 Introduction

In human embryonic development, cortical neuron differentiation is a highly intricate process that progresses through a series of tightly regulated stages, generating multiple intermediate cell types. This journey begins with gastrulation around day 14 post‐fertilization, when the epiblasts develop into ectoderm.^[^ ^1^, ^2^, ^3^, ^4^ ^]^ From the ectoderm, neuroepithelial (NE) cells arise and proliferate to form the neural plate by days 18–20, which then closes to form the neural tube by day 28.^[^ ^3^ ^]^ Cortical development starts with neurogenesis from neural progenitor cells (NPC) derived from NE cells in the ventricular zone.^[^ ^3^, ^5^ ^]^ Neurogenesis in the developing cortex follows a predictable timetable,^[^ ^6^ ^]^ beginning around day 42 and continuing until around week 21, with different cortical layers forming sequentially.^[^ ^7^, ^8^, ^9^ ^]^ This process involves radial glial (RG) cells and intermediate progenitors (IP), with RG cells undergoing direct or indirect neurogenesis to produce distinct neuronal subtypes.^[^ ^10^, ^11^, ^12^, ^13^ ^]^ Maturation of cortical neurons extends through the second and third trimesters, involving axon and dendrite growth, synapse formation, and expression of specific neurotransmitters and receptors.^[^ ^14^, ^15^, ^16^ ^]^

Forward genetic screening approaches have revealed roles for both epigenetic modifiers and transcription factors in the regulation of a cell‐intrinsic clock that coordinates the timing of neurogenesis and the fate of progenitor cells.^[^ ^17^, ^18^ ^]^ At the molecular level, this process is coordinated by an extensively characterized regulatory circuitry involving dynamic oscillatory and temporal expression of NOTCH signaling components and proneuronal basic helix‐loop‐helix (bHLH) transcription factors (TFs).^[^ ^19^ ^]^ The oscillations in *HES* genes (e.g., *HES1*, *HES5*) and proneuronal genes (e.g., *NEUROG1*, *ASCL1*),^[^ ^20^ ^]^ modulated by mutual inhibition, maintain the population of neural progenitor cells.^[^ ^21^, ^22^, ^23^ ^]^ Termination of these oscillatory dynamics results in sustained elevation of proneuronal transcription factor expression, triggering cell cycle exit and initiation of neuronal differentiation programs.^[^ ^22^, ^23^ ^]^ Through these precisely timed molecular interactions, the regulatory network progressively constrains progenitor developmental potential while establishing layer‐specific neuronal identity by modulating the temporal kinetics of neurogenic commitment.^[^ ^24^, ^25^ ^]^

To mimic the intricate developmental sequence of cortical neurons in vitro, researchers have developed methods to differentiate human pluripotent stem cells (hPSCs) into cortical neurons. These methods utilize signaling molecules, such as those employed in DS inhibition, to guide hPSCs through a stepwise differentiation trajectory.^[^ ^26^, ^27^, ^28^, ^29^ ^]^ These in vitro systems not only generate functionally mature neurons but also faithfully recapitulate key aspects of in vivo developmental progression, albeit on an accelerated schedule.^[^ ^30^ ^]^ Despite this acceleration, the processes remain time‐intensive, often taking several weeks for neurogenesis and months to produce mature neuronal populations.^[^ ^26^, ^31^ ^]^

Alternatively, a more expedient strategy employs proneuronal transcription factors (TFs), such as NEUROG2 or ASCL1, to directly program hPSCs into neurons.^[^ ^32^, ^33^, ^34^, ^35^, ^36^ ^]^ We and many others have observed a striking divergence in neurogenesis kinetics between TF‐induced forward programming and DS inhibition.^[^ ^30^, ^37^ ^]^ TF‐induced forward programming rapidly yielded bipolar neuronal morphology with >90% purity within 4 days, whereas DS inhibition required several weeks to achieve similar neuronal yields.^[^ ^30^, ^38^ ^]^ Intriguingly, despite this substantial temporal disparity in neurogenesis, the subsequent maturation rate of the resulting neurons remains comparable. Previous studies have indicated that TF‐induced cells traverse a distinct, naturally unfound intermediate state, ultimately reaching a functional state akin to that induced by DS inhibition.^[^ ^35^, ^37^ ^]^ Although a number of studies have provided insights into the stepwise transcriptional and chromatin state changes underlying TF‐induced forward programming,^[^ ^32^, ^39^, ^40^, ^41^, ^42^, ^43^, ^44^ ^]^ the mechanism by which proneuronal TF overexpression accelerates cell fate commitment and neurogenesis remains obscure. This critical gap in understanding drives our investigation into whether TF‐induced programming can be harnessed to enhance in vitro neuronal maturation—a vital step for modeling neurological disorders characterized by mature neural circuit dysfunction.

In this study, we reconstructed the molecular trajectories and identified distinct intermediate progenitors involved in two differentiation strategies of hPSCs. We used multi‐omic analysis to identify potential regulatory mechanisms by which three proneuronal TFs promote a more defined and rapid specification of glutamatergic neurons. Moreover, we dissected the gene regulatory networks (GRNs) that modulate the timing of neurogenesis and maturation. Our findings hold promise of advancing our understanding of the transcriptional mechanisms governing cell fate determination and temporal control during human cortical development, with implications for the rational design of time‐controlled protocols for generating precise neuronal subtypes from hPSCs and for therapeutic applications in neurodevelopmental disorders.

## 2 Results

### 2.1 scRNA‐seq Over the Time Course of Neuronal Differentiation From hPSCs Revealed Diverging Trajectories

**Figure 1:**
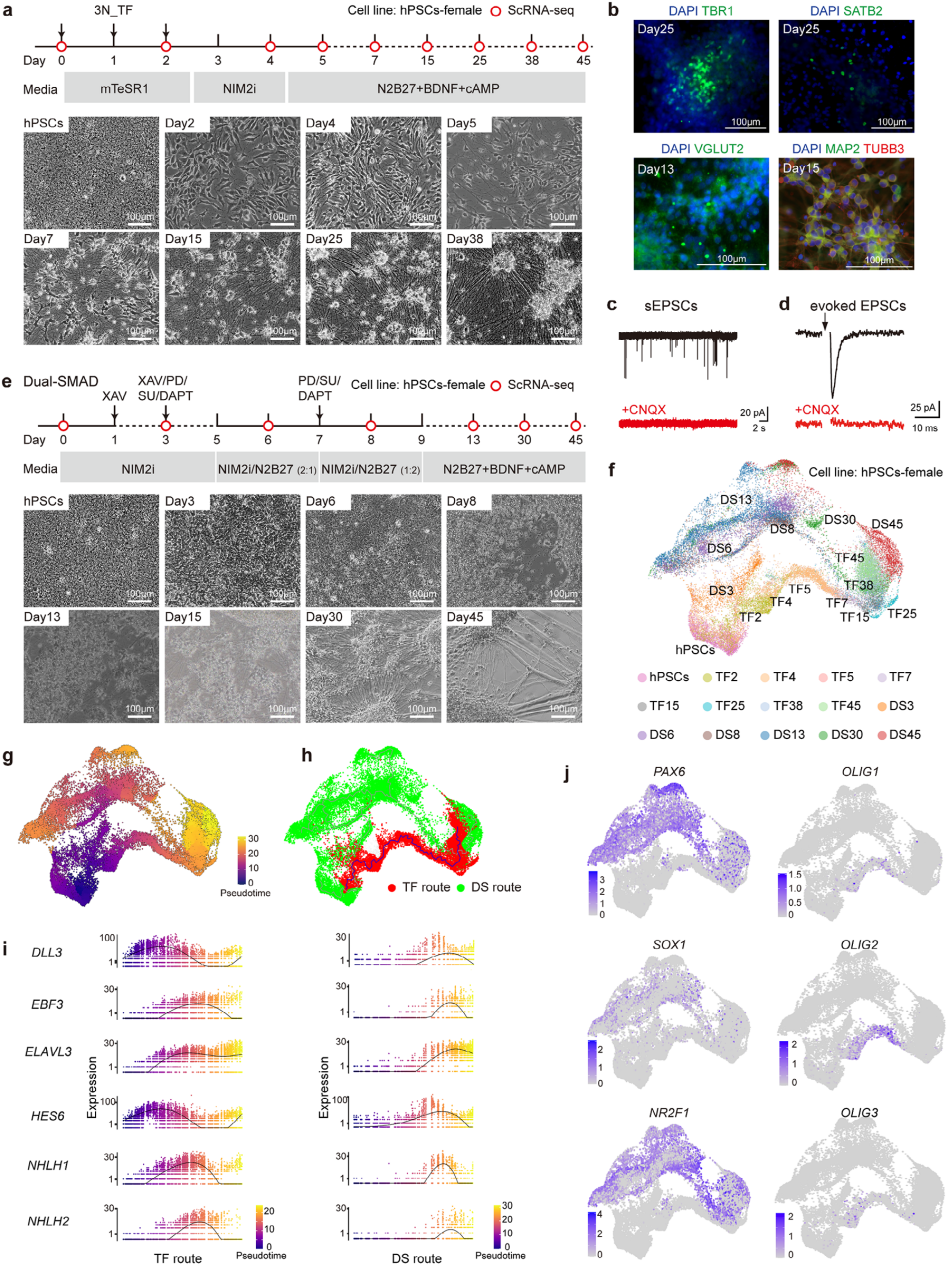
Single cell RNA‐seq (scRNA‐seq) reconstruction of neuronal differentiation from human pluripotent stem cells (hPSCs). a) Schematic of TF‐induced forward programming. After seeding hPSCs at day 0, 3N_TF are transfected three times in the form of synthetic mRNAs from day 0 to day 2. Differentiation media used and cellular morphology during differentiation are indicated below. Time points at which cells were harvested for scRNA‐seq profiling were marked with red circles. hPSCs‐female cell line was used for neuron induction. Scale bars are 100 µm. b) Expression of neuronal markers (TBR1, SATB2, VGLUT2, TUBB3, and MAP2) in differentiating cells. TBR1, preplate, subplate, and cortical layer VI neuron marker; SATB2 (layer II–III, V); VGLUT2, glutamatergic neuron marker. Scale bars are 100 µm. c,d) Representative traces of spontaneous excitatory postsynaptic currents (sEPSCs) (c) and evoked EPSCs (d) recorded in 3N_TF induced neurons without inhibitors at day 38. Both sEPSCs and evoked EPSCs signals can be blocked by CNQX, a competitive antagonist of AMPA glutamate receptors. e) Schematics of DS inhibition. Differentiation media used and cellular morphology during differentiation are indicated below. Time points at which cells were harvested for scRNA‐seq profiling are marked with red circles. hPSCs‐female cell line was used for this analysis. Scale bars are 100 µm. f) Uniform manifold approximation and projection (UMAP) plot of all the cells from different time points. TF represents cells differentiated by 3N_TF, and DS represents cells differentiated by DS inhibition. g) Pseudotime analysis by Monocle 3. Cells are labeled by pseudotime values. h) Cellular trajectory reconstruction using Monocle3. TF‐induced forward programming cells are colored in red (the TF route), and cells induced by DS inhibition are colored green (the DS route). Blue and grey lines on the UMAP plots represent the trajectory graph. i) Differential expression of neural related genes (*ELAVL3*, *EBF3*, *NHLH1*, and *NHLH2*) and NOTCH signal pathway related genes (*DLL3* and *HES6*) in the TF and the DS route with pseudotime scale. The color scale bar indicates the values of pseudotime. j) UMAPs depicting the expression of *PAX6*, *SOX1*, *NR2F1*, *OLIG1*, *OLIG2*, and *OLIG3* expression.

We sought to develop an efficient and integration‐free synthetic mRNA‐based strategy to forward program hPSCs to glutamatergic neurons. For this purpose, we began by combining six well‐established TFs crucial for neurogenesis: NEUROG1, NEUROG2, NEUROG3, NEUROD1, NEUROD2, and ASCL1.^[^ ^32^, ^41^, ^45^, ^46^ ^]^ Following introduction into hPSCs, we observed rapid morphological changes in the cells, with the emergence of neuron‐like cells just three days after transfection. By day 7, most cells appeared as mature neurons with long neural processes (Figure S1a, Supporting Information). To refine the number of TFs needed for effective neuron generation, we systematically removed each factor from our initial combination and quantified the percentage of cells with long neural processes that were positive for β‐III‐tubulin TUBB3 staining. We observed that exclusion of NEUROG1, NEUROG2, or NEUROD1 resulted in a decreased percentage of neurons, while the removal of NEUROG3 led to an increased number of neurons (Figure S1b, Supporting Information). In contrast, the absence of NEUROD2 or ASCL1 did not significantly affect neurogenesis efficiency. Based on these results, we used the TF combination, of NEUROG1, NEUROG2, and NEUROD1, which we refer to as “3N_TF” for subsequent experiments. Concurrently, we also tested five different culture media to enhance differentiation (Stem Fit, NSC, NPC, Neuron, and NIM2i). We found that NIM2i media, which contains both BMP and TGF‐β inhibitors, yielded the highest percentage of neurons, as indicated by TUBB3 and MAP2 staining (Figure S1c,d, Supporting Information). With the optimization of TF combinations and culture conditions, we observed neurons within 4 days, with > 95% of cells expressing TUBB3 and MAP2 (**Figures** **1**a, S1e, Supporting Information). Resulting neurons expressed proteins specific to glutamatergic neurons (marked by TBR1, SATB2, and VGLUT2)^[^ ^47^ ^]^ (Figure 1b) and also exhibited transcriptional characteristics of glutamatergic neurons (Figure S1f, Supporting Information). Electrophysiological analysis revealed that all recorded cells generated action potentials and active membrane properties after hyperpolarization (Figure S1g–m, Supporting Information). In the presence of picrotoxin, a GABA_A receptor antagonist, bursting of spontaneous excitatory postsynaptic currents (EPSCs) was observed and could be blocked by CNQX (an AMPA (a‐amino‐3‐hydroxy‐5‐methyl‐4‐isoxazole propionic acid) receptor blocker), indicating the expression of functional excitatory neurotransmitter systems (Figure 1c). Picrotoxin was used to eliminate potential noise from GABAergic neurons, which prior studies indicate may be generated in TF‐induced differentiation systems.^[^ ^48^, ^49^ ^]^ Focal stimulation evoked EPSCs were also able to be blocked by CNQX (Figure 1d), indicating the cells form functional synapse.

We next sought to compare differentiation trajectories of TF‐induced forward programming with an accelerated DS differentiation protocol, which incorporates mitotic inhibitors (PD0325901, SU5402, and DAPT) to enhance neuronal differentiation. Previous scRNA‐seq analyses have highlighted distinct differentiation trajectories in mouse motor neuron differentiation resulting from TF‐induced forward programming and stepwise cellular differentiation.^[^ ^37^ ^]^ However, a comprehensive comparison of these differentiation trajectories in the context of human glutamatergic neuron differentiation, as outlined here, particularly with the aim of analyzing differential differentiation speeds, has not been reported. Thus, we performed scRNA‐seq on cells over the time course of TF‐induced forward programming and stepwise cellular differentiation (Figure 1a,e). By combining all the time‐course data from a total of 22 947 cells, we reconstructed continuous differentiation trajectories from pluripotency to neurons for both TF‐induced forward programming and DS inhibition (Figure 1f). The cells were organized based on differentiation pseudo‐time, revealing two distinct branches leading from their respective starting points to the end points (Figure 1g). Further analysis using the Monocle3 algorithm, which enables the use of scRNA‐seq data for trajectory analysis, confirmed the presence of two diverging trajectories for TF‐induced forward programming (referred to as the TF route) relative to DS inhibition (referred to as the DS route) (Figure 1h). Notably, we observed that genes essential for neuronal differentiation (*DLL3*, *HES6*, *EBF3*, *ELAVL3*, *NHLH1*, and *NHLH2*)^[^ ^50^, ^51^, ^52^, ^53^, ^54^, ^55^, ^56^ ^]^ were induced and expressed with different kinetic profiles (Figure 1i). Specifically, classic neural progenitor marker genes, such as *PAX6*, *SOX1*, and *NR2F1*,^[^ ^57^, ^58^ ^]^ are exclusively expressed in the DS route (Figure 1j; Figure S2a, Supporting Information), whereas other genes, such as *OLIG2*, and *OLIG3*, which are associated with oligodendrocyte precursors and motor neuron development,^[^ ^59^, ^60^, ^61^ ^]^ were specifically expressed in the TF route. These results indicate that hPSCs differentiating to neurons via the TF route go through distinct progenitor stages independent of *SOX1* and *PAX6*. To assess in vivo relevance, we compared TF‐ and DS‐derived scRNA‐seq data with a developing human cortex dataset (GSE155121, 431080 cells, seven samples, Carnegie stages 10‐20, 3‐8 weeks). Both TF‐ and DS‐derived endpoint neurons exhibited strong transcriptional correlation with cortical glutamatergic neurons, indicating that our in vitro differentiation protocols recapitulate critical aspects of in vivo cortical neuron development (Figure S2b,c, Supporting Information).

To exclude the possibility of potential heterogeneity caused by variations in synthetic mRNA dosage within individual cells, we generated a knock‐in doxycycline‐inducible system to endogenously express 3N_TF under the control of doxycycline in male and female hPSCs lines (dox‐inducible 3N_TF OE hPSCs line) (Figure S2d‐f, Supporting Information). Time course analysis of scRNA‐seq data further confirmed the induction of the TF route in this system as well (Figure S2e‐g, Supporting Information). Taken together, these data indicate that TF‐induced forward programming represents a distinct differentiation shortcut from hPSCs to neuronal fate.

### 2.2 Deciphering Transcriptional Trajectories Revealed Dynamics of Neuronal Differentiation

**Figure 2:**
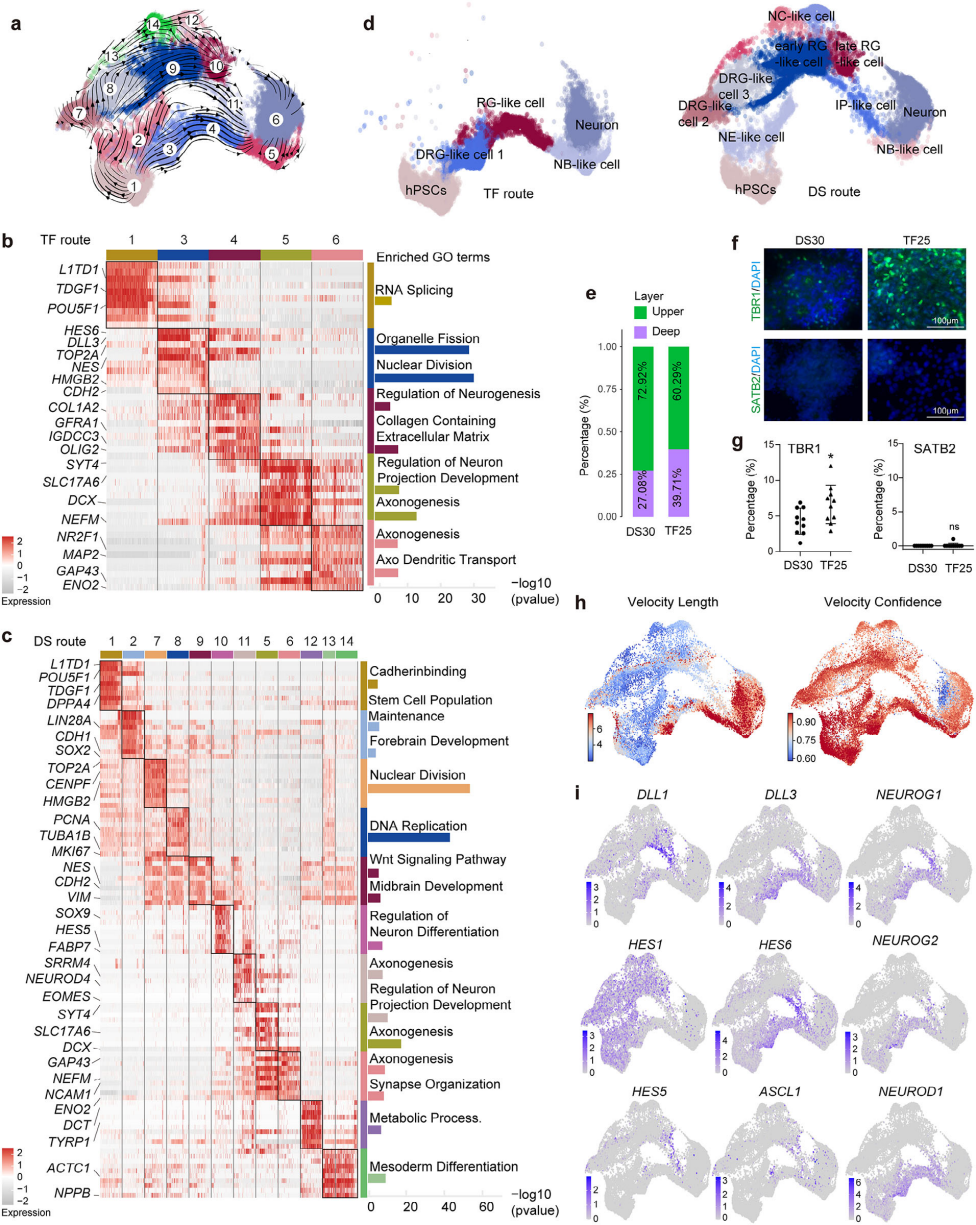
Transcriptomic analyzes of molecular mechanisms underlying different rates of neuronal differentiation. a) Estimated RNA velocity field in streamlines by scVelo. Cells are divided into 14 clusters based on their transcriptomes and indicated by different colors. b,c) Heatmap showing the mean expression of marker genes of the major clusters in the TF (b) and the DS route (c). Enriched gene ontology (GO) terms for the top100 marker genes of the major clusters (black boxes) are shown vertically on the right, and the bar graph shows the negative logarithmic p‐values. d) Annotation of the clusters by the expression of marker genes. hPSCs, induced pluripotent stem cells; NE, neuroepithelial; RG, radial glia; NB, neuroblast; IP, intermediate progenitor; DRG, dividing radial glia; NC, neural crest. e) Stacked bar plot illustrating the proportions of upper‐ and deep‐ neurons. f) Representative immunofluorescence images for TBR1 and SATB2 in 3N_TF and DS inhibition induced neurons. Scale bars are 100 µm. hPSCs‐male cell line was used. g) Quantification of TBR1^+^ and SATB2^+^ cell percentage. Data are presented as mean ± SD of n = 3 independent replicates. *p < 0.05; **p < 0.01; ***p < 0.001; ****p < 0.0001. h) Velocity analysis showing the estimated differentiation speed by software scVelo. The estimated differentiation speed is given by the length of velocity vector and the velocity confidence represents the measure of confidence. i) UMAPs depicting the expression of *DLL1*, *DLL3*, *HES1*, *HES6*, *HES5*, *ASCL1*, *NEUROG1*, *NEURG2*, and *NEUROD1* expression. The color scale bar indicates the level of gene expression. *DLL1*, *HES1*, and *HES5* are genes known to inhibit neurogenesis; *DLL3*, *HES6*, and *ASCL1* are proneural genes.

To further probe the transcription programs underlying the two differentiation routes, we clustered the cells based on their gene expression profiles and performed RNA velocity analysis. The unsupervised clustering identified 14 distinct clusters, and subsequent RNA velocity in single cells demonstrated two continuous directional flows. These flows originated from cluster 1, enriched with expression of pluripotency genes, progressing toward cluster 6, characterized by the expression of mature neuron genes (**Figure** **2**a). Specifically, cells following the TF route underwent a rapid cell fate transition through clusters 3, 4, and 5 (Figure 2b). In contrast, cells following the DS route experienced a more complex gene expression pattern across clusters 2, 7, 8, 9, 10, and 11 (Figure 2c).

We further annotated cells within each trajectory based on the expression of classic marker genes (Figure 2b–d). This revealed that the DS route recapitulated a progressive differentiation trajectory that mirrored in vivo development. In the DS route, hPSCs differentiated into neuroepithelial‐like (NE‐like) cell (cluster 2, marked by *CDH1* and *SOX2*)^[^ ^62^, ^63^, ^64^ ^]^ within 3‐5 days, aligning with the in vivo emergence of NE cells from the ectoderm. By around day 12, these NE‐like cells transitioned into dividing radial glia‐like (DRG‐like) cells (cluster 7 and 8, marked by *MKI67*, *TOP2A*),^[^ ^65^ ^]^ followed by radial glia‐like (RG‐like) cells (cluster 9 annotated as early RG‐like cells and marked by *NES, CDH2, VIM*;^[^ ^63^, ^64^, ^66^ ^]^ cluster 10 annotated as late RG‐like cells and marked by *FABP7, HES5*, and *SOX9*).^[^ ^64^, ^66^, ^67^, ^68^ ^]^ Between days 25 and 30, late RG cells differentiated into intermediate progenitor‐like (IP‐like) cells (cluster 11, marked by *EOMES*)^[^ ^65^, ^69^, ^70^ ^]^ and initiate neurogenesis, producing neuroblast‐like (NB‐like) cells (cluster 5, marked by *DCX*),^[^ ^71^, ^72^ ^]^ ‐corresponding to the in vivo onset of cortical neurogenesis at day 42 post‐fertilization. These NB‐like cells then required an additional 15–30 days to develop into mature neurons (cluster 6, marked by *MAP2, NEFM, GAP43, ENO2*) in vitro.^[^ ^73^, ^74^, ^75^, ^76^, ^77^, ^78^ ^]^ While DS‐induced differentiation generates heterogeneous populations, including transient neural crest‐derived cells in clusters 12, 13, and 14, these non‐neuronal cell types do not persist in the final neuronal culture due to selective culture conditions.

In contrast, the TF route bypassed the NE stage and exhibited commitment to a distinct type of RG cells (clusters 3 and 4), marked by *TOP2A, NES, CDH2, DLL3*, and *OLIG2*. This route‐initiated neurogenesis within the first 48 h and then proceeded directly to differentiate into NB‐like cells and mature neurons, skipping the IP stage. The trajectory analyses revealed two acceleration steps: First, initial commitment to neuronal fate, bypassing the NE stage, and second, direct neurogenesis from RG cells, skipping the IP stage. We verified the scRNA‐seq results through morphological and immunostaining analyses. In the DS route, cells displayed prolonged epithelial morphology and expressed *CDH1*, an NE marker gene, for approximately 4 days until the formation of neural rosettes and the activation of *CDH2*, a marker gene for RG cells (Figure 1e, Figure S2h,i). However, overexpression of 3N_TF caused colony‐shaped hPSCs to spread out and adopt a thin and elongated cell, deviating from the typical tight cell‐cell junction morphology (Figure 1a, Extended Data Video S1, Supporting Information). Immunostaining confirmed the loss of CDH1 protein in differentiating cells that spread out from the colony on day 1, further supporting the bypass of the NE stage (Figure S2h,I, Supporting Information). Additionally, scRNA‐seq and immunostaining revealed that cells in the DS route express *EOMES*, a marker gene for IPs, while *EOMES* is absent in the TF route (Figure S2j,k, Supporting Information). We further analyzed layer‐specific identities of neurons using module scores derived from scRNA‐seq expression of genes associated with deep‐ (*FEZF2*,^[^ ^79^, ^80^ ^]^ *BCL11B*,^[^ ^79^, ^81^, ^82^ ^]^ *SOX5*,^[^ ^79^ ^]^ *NFIA*,^[^ ^83^ ^]^ *NFIB*,^[^ ^83^ ^]^ *CELF1*,^[^ ^84^ ^]^ *TLE4* ^[^ ^81^ ^]^ *and FOXP2* ^[^ ^80^, ^82^ ^]^) or upper‐layer (*MEF2C*,^[^ ^79^ ^]^ *RORB*,^[^ ^80^ ^]^ *SATB2*,^[^ ^79^, ^81^ ^]^ *CUX1*,^[^ ^49^, ^80^, ^85^ ^]^ *CUX2* ^[^ ^79^, ^85^ ^]^ *and POU3F2* ^[^ ^79^ ^]^) cortical neurons, complemented by immunostaining quantification. These results demonstrate that TF‐induced differentiation, by bypassing IPs, yields a higher proportion of deep‐layer cortical neurons compared to the DS route (Figure 2e–g).^[^ ^86^ ^]^ These findings suggest that RG cells derived from both routes undergo different neurogenesis pathways, ultimately generating neurons of distinct cortical subtypes: the DS route reflects an indirect neurogenesis process involving IPs, whereas the TF route mimics direct neurogenesis.

Next, we conducted velocity analysis and identified distinct velocity patterns of neurogenesis in the two routes. While cells on the DS route underwent slow transitions from cluster 1 to cluster 6, cells on the TF route exhibited a rapid cell fate transition through a shortcut pathway (Figure 2h). In light of the known role of NOTCH signaling components in regulating the pace of neurogenesis in animal models,^[^ ^19^, ^87^, ^88^ ^]^ we sought to examine the expression of genes related to NOTCH signaling. Results demonstrated that *HES1, HES5*, and *DLL1*, all of which suppress the expression of proneuronal genes and inhibit neurogenesis,^[^ ^23^, ^88^, ^89^ ^]^ were highly expressed in the DS route. Conversely, genes that promote neurogenesis, such as *HES6*, *DLL3*, and *ASCL1* ^[^ ^50^, ^52^, ^90^, ^91^ ^]^ were repressed in the DS route until the induction of endogenous *NEUROG1*, *NEUROG2*, and *NEUROD1* (Figure 2i). When we ectopically expressed 3N_TF in hPSCs, the expression of *HES1* and *DLL1* was repressed, subsequently triggering the immediate induction of proneuronal genes in the TF route (Figure 2i). Collectively, these findings support the notion that neurogenesis in the DS route is prolonged due to the mutual inhibition of NOTCH signals and proneuronal genes; conversely, neurogenesis in the TF route is rapidly induced through the activation of proneuronal genes.

In summary, the transcriptomic analyses provide insight into the drastic cell fate conversion induced by 3N_TF, which can be characterized by two distinct acceleration steps: initial commitment to neuronal fate, bypassing the NE stage; followed by direct neurogenesis from RG cells without mutual inhibition of NOTCH signals and proneuronal genes.

### 2.3 Resolution of the Gene Regulatory Cascades Driving Rapid Cell Fate Commitment Through High‐Resolution Multi‐Omics Analysis

**Figure 3:**
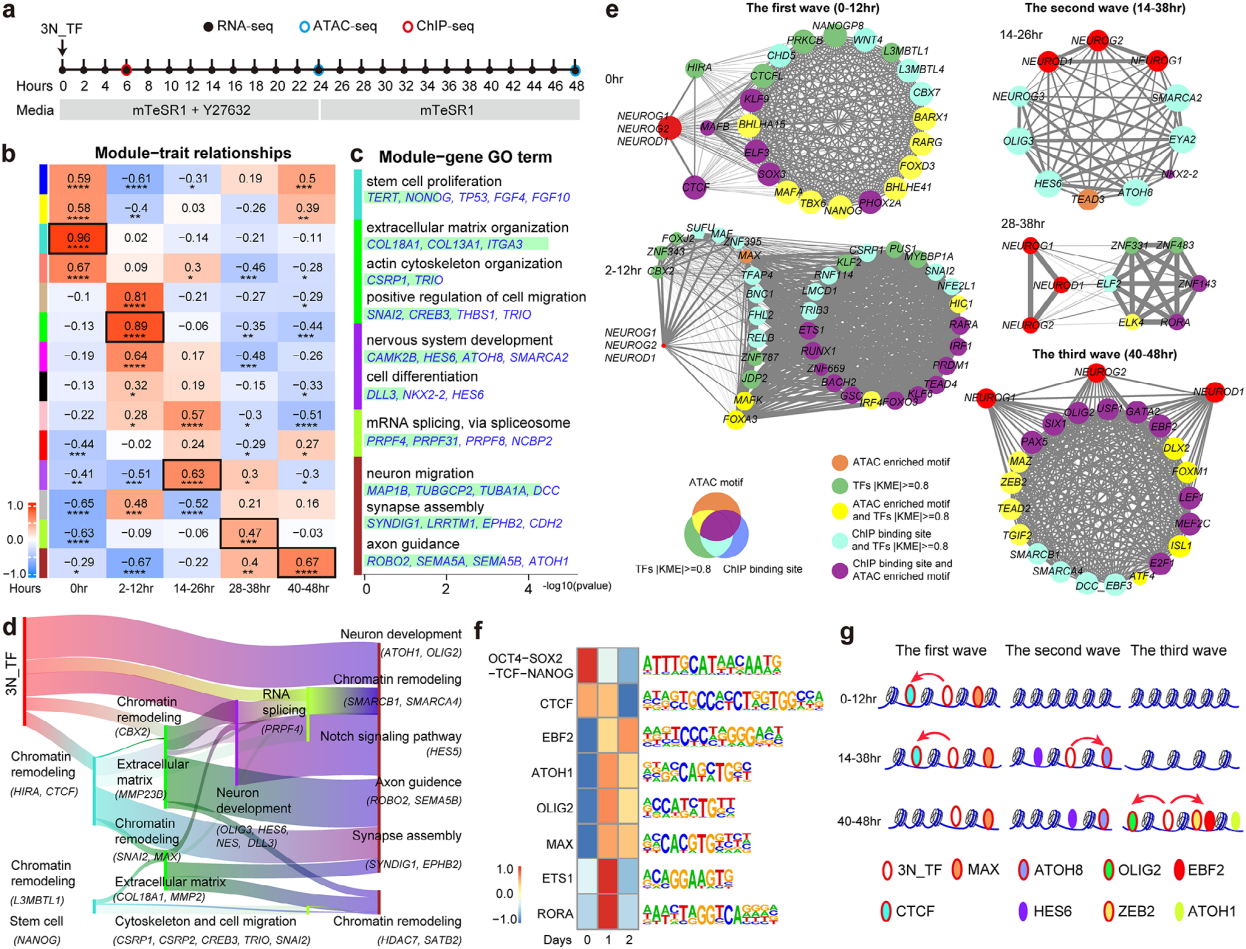
High‐resolution multi‐omics analysis of gene regulatory cascades that drive cell fate commitment in human neurogenesis in vitro. a) Schematic overview of the experimental procedure for multi‐omics analyses. Following the induction of neurons with 3N_TF, cells were harvested to conduct RNA‐seq (every 2 h within the first 48 h), ATAC‐seq (at 0, 24, and 48 h), and ChIP‐seq (NEUROG1 ChIP‐seq, NEUROG2 ChIP‐seq, NEUROD1 ChIP‐seq at 6 h). h, hour. hPSCs‐female cell line was used for neuron induction. b) Identification of co‐expression modules by weighted gene co‐expression network analysis (WGCNA). The heatmap shows the correlation of the identified modules with the five states of neuronal differentiation. According to the correlation value, five modules with the highest correlation value at each state were selected for further analysis (labeled by the black boxes). c) Enriched gene ontology (GO) terms for the module genes (black boxes) and the bar graph showing the negative logarithmic p‐values. d) Sankey plots presenting the correlation and the regulatory cascades of TFs. The width of the lines representing the correlation between the two modules. e) Transcription factor regulatory network analysis for five molecular states. The size of the nodes represents the quantity of associations, and the width of the lines represents the correlation between the two TFs. We selected TFs with KME>=0.8 or/and TFs that bound by 3N_TF or/and TFs whose motifs are enriched in ATAC datasets as input genes for visualization by CytoScape. The nodes were colored based on the features of TFs. f) Heatmap showing the relative frequencies of known results motif identified by ATAC‐seq analysis. g) Proposed model of the regulatory cascade during TF‐induced forward programming. 3N_TFs, in conjunction with TFs activated in the early phase, directly interact with regulatory regions of crucial TFs involved in neuronal differentiation, resulting in a rapid cascade of gene expression and cell fate conversion. Initially, 3N_TF initiate activation of chromatin remodelers, such as CTCF and MAX, to modify chromatin accessibility of downstream TFs. Subsequently, 3N_TFs bind to regulatory regions of a few proneuronal TFs (e.g., ATOH8, HES6) to trigger their expression. These TFs, in collaboration with the 3N_TF, then facilitate activation of numerous downstream TFs, including additional chromatin remodeling TFs and proneuronal TFs (e.g., OLIG2, ZEB2, ATOH1, EBF2).

As we noted above that neurogenesis was initiated in the TF route within the first 48hrs, we sought to further decipher the regulatory landscape underlying the drastic cell fate changes by high‐resolution time‐course RNA expression profiling (every 2 h within the first 48 h), chromatin accessibility analysis (ATAC analysis at 0, 24, and 48 h), and TF binding profiling by CUT‐TAG (NEUROG1, NEUROG2, and NEUROD1 CUT‐TAG at 6 h) (**Figures** **3**a, S3a–c, Supporting Information). Leveraging a non‐negative matrix factorization algorithm, we identified five distinct molecular states that guide progressive cell fate transition based on the dynamic of transcriptomes (0, 2–12, 14–26, 28–38, and 40–48 h) (Figure S3d,e, Supporting Information). Furthermore, weighted gene co‐expression network analysis (WGCNA) allowed us to identify co‐expression modules within each cluster (Figures 3b, S3f, Supporting Information). Gene ontology (GO) analysis of the highly correlated genes within each cluster revealed a cascade of molecular events leading to rapid neurogenesis. Upon activation of 3N_TF, the gene expression profile shifted immediately from “stem cell proliferation” to the activation of chromatin remodeling genes (e.g., *HIRA*, *CTCF*, and *CBX7*) (Figures 3d, S3f,h, Supporting Information, the turquoise module). In response to the reshaping of the chromatin landscape, a series of genes were co‐expressed concurrent with the reorganization of the extracellular matrix (e.g., *COL18A1*, *COL13A1*), cytoskeleton (e.g., *CSRP1*, *TRIO*), as well as cell shape transitions and cellular migration (e.g., *CREB3*, *SNAI2*) (Figure 3c,d, the green module). These results correspond with the rapid loss of epithelial morphology and cell spreading observed on day 1 of differentiation (Figures 1a, S2d, Supporting Information). Notably, during the first 12 hours, cells extended filopodia and lamellipodia‐like projections and migrated (Figure S3g, Supporting Information). Moreover, *ZEB2*, which functions to promote cell shape transition to RG cells,^[^ ^92^ ^]^ and *SNAI2*, which regulates neuronal migration ^[^ ^93^ ^]^ were both activated concurrently with the morphological change toward a neuronal fate (Figures 3c, S3g, Supporting Information). Subsequently, another wave of neural differentiation‐related TFs, such as *HES6*, *NEUROG3*, and *ATOH8*, was activated (Figures 3c, S3h, the purple module). Following this, genes associated with mRNA splicing (e.g., *PRPF4*, *NCBP2*) were induced, profoundly shaping the alternative splicing pattern (Figure 3c, the green‐yellow module), potentially mimicking splicing switches during neural development in vivo.^[^ ^94^ ^]^ At a later stage (40–48 h), 3N_TF promoted the expression of genes related to synapse assembly and axon maturation (e.g., *ATOH1*, *CDH2*, and *ROBO2*) (Figure 3c,d, the brown module). Fuzzy clustering analyses further demonstrated the dynamic expression of genes, providing insights into the regulatory hierarchy governing this rapid cell fate transition (Figure S3i,j, Supporting Information).

To uncover the regulatory hierarchy underlying cell fate commitment, we dissected TF regulatory networks based on ATAC‐seq and CUT‐TAG data and observed that the 3N_TF extensively co‐bind the regulatory regions of genes in the genome (Figure S3a‐c,k, Supporting Information). We performed multi‐omics analysis, focusing on TFs that were directly activated by 3N_TF and thus might potentially function as effectors to change the global expression profiles. We observed that overexpression of 3N_TF directly activated three waves of downstream TFs, including several identified as neuron‐essential TFs,^[^ ^41^ ^]^ within 48 h. The first wave (0–12 h) involved the induction of chromatin remodeling and organization TFs (e.g., *CTCF, CHD5*), leading to the exit from the pluripotency program.^[^ ^95^, ^96^ ^]^ The second wave encompassed the activation of neurogenesis TFs, such as *HES6, ATOH8, and NEUROG3*, initiating neuronal‐specific expression profiles (14–38 h).^[^ ^97^, ^98^ ^]^ In the final wave (38‐48 h), more chromatin remodeling (e.g., *SMARCA4, SMARCB1*) and proneuronal TFs, including *OLIG* family TFs (*OLIG1, OLIG2*), were activated to promote comprehensive cellular differentiation towards neurons (Figure 3e). Additionally, key TFs were found to extensively engage distal and proximal regulatory regions to regulate global gene expression, as indicated by overrepresented DNA sequence motif searching (Figure 3f). These findings provide insights into a distinct gene regulatory cascade underlying transcription factor‐induced cell fate determination (Figure 3g).

### 2.4 NEUROG1 and NEUROD1 Co‐Expression Modulated NEUROG2 Binding for Enhanced Neuronal Identity Specification

**Figure 4:**
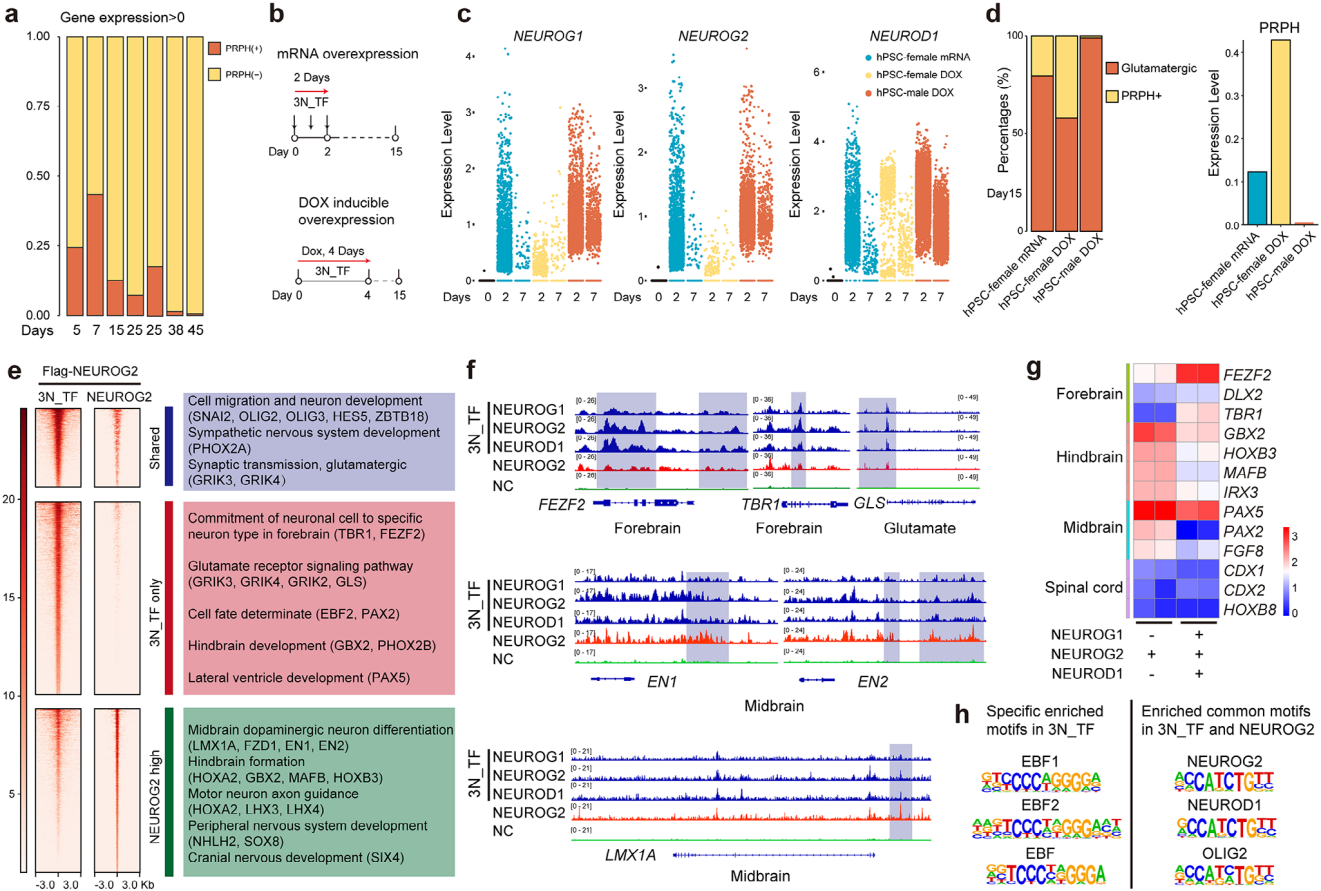
NEUROG1 and NEUROD1 modulate chromatin targeting, promote differentiation of forebrain neurons, and repress heterogeneity of neurons induced by NEUROG2. a) Bar plot illustrating the proportions of PRPH^+^ and PRPH^−^ cells induced by 3N_TFs from day 5 to 45. PRPH^+^ cells were identified based on gene expression levels (PRPH) > 0. b) Schematic illustrating the overexpression of 3N_TF using mRNA or doxycycline treatment in the experimental paradigm. Two hPSCs cell lines (hPSCs‐female and hPSCs‐male) were used. c) The expression levels of *NEUROG1*, *NEUROG2*, and *NEUROD1* detected in scRNA‐seq analysis 3N_TF across different samples 3N_TF. d) Stacked bar plot illustrating the proportions of cell types and the expression levels of PRPH as determined by scRNA‐seq. e) Left: ChIP‐seq profile of flag‐NEUROG2 within ±3 kb from the peak center (red signal) in hPSCs, 6 h after transfection with flag‐NEUROG2 alone (labeled as NEUROG2) or in combination with NEUROG1 and NEUROD1 (labeled as 3N_TF). A total of 3465 shared peaks were identified using the mergePeaks function with default parameters. Right: Gene ontology terms for various genomic clusters deemed significant by DAVID analysis, based on genes located within 3000 kb from the peaks. f) Genome browser visualization of the 3N_TFs binding tracks within several gene loci. g) Heatmap showing the expression of neural genes representing the forebrain, midbrain, hindbrain, and spinal cord regions in cells induced by NEUROG2 alone (data obtained from GSE181019) and 3N_TFs at day2. h) Top enriched motifs identified by ChIP‐seq analysis in each context.

Forced expression of NEUROG2 in hPSCs has been reported to induce a spectrum of neuronal subtypes, including those that express PRPH, a marker for the peripheral nervous system.^[^ ^35^, ^99^ ^]^ To examine the consequences of NEUROG1 and NEUROD1 co‐expression, we first investigated the heterogeneity of our endpoint cells. We observed that the endpoint cells from both the TF and DS route were primarily composed of glutamatergic neurons, with a smaller proportion of GABAergic and peripheral neurons (Figure S4a‐d, Supporting Information). Consistent with published data, DS inhibition resulted in a mixed population of glutamatergic and GABAergic neurons.^[^ ^17^, ^100^, ^101^ ^]^ In contrast, TF‐induced forward programming produced a more homogenous population predominantly composed of glutamatergic neurons (Figure S4e,f, Supporting Information). During early TF‐induced differentiation, a transient PRPH+ population, co‐expressing glutamatergic markers (e.g., *SLC17A6*, *GLS*), was observed (Figure S4b,j, Supporting Information). This population significantly diminished over time, transitioning into glutamatergic neurons, indicating an intermediate state rather than a committed peripheral nervous system (PNS) fate (Figure S4a–j, Supporting Information). This shift reflects the emergence of a homogeneous cortical neuron population. Notably, the extent of this heterogeneity closely correlated with the expression dosages and duration of exogenous 3N_TF. The doxycycline‐inducible male hPSCs line, characterized by the highest expression and duration of 3N_TF, exhibited the lowest percentage of PRPH+ cells, indicating a more homogeneous differentiation outcome (**Figure** **4**b–d, Figure S4g‐j, Supporting Information). These results indicate that co‐expression of bHLH TF partners with NEUROG2 enhances neuronal identity specification in glutamatergic neurons, and the differentiation efficiency correlates with the dosages and duration of the exogenous genes.

To explore mechanisms by which NEUROG1 and NEUROD1 restrict NEUROG2's ability to induce neuronal subtypes, we compared the genomic binding of NEUROG2 in the presence and absence of NEUROG1 and NEUROD1. Interestingly, the addition of NEUROG1 and NEUROD1 significantly redistributes the genome bindings of NEUROG2, modulating not only the intensity of shared NEUROG2 binding sites but also cooperating with NEUROG2 to bind to a multitude of new genomic loci (Figure 4e). This redistribution results in the activation of glutamatergic neuron genes, while simultaneously repressing the expression of non‐glutamatergic gene programs (Figure 4f,g). *De novo* motif search analyses indicated that binding sites exclusive to the 3N_TF were enriched for motifs corresponding to EBF family TFs (Figure 4h). As demonstrated in our scRNA‐seq results, EBF family TFs were expressed in the later stages of neurogenesis in both the TF and DS route (Figure 1i, Figure S4k, Supporting Information). Thus, our data indicate that the combination of NEUROG1 and NEUROD1 with NEUROG2 restricts neuronal differentiation towards the glutamatergic program by cooperatively enhancing its activation on genes that define glutamatergic subtypes.

### 2.5 Analysis of Transcriptional Modules in the TF Route Revealed Distinct Gene Regulatory Networks (GRNs) for Neurogenesis and Neuronal Maturation

**Figure 5:**
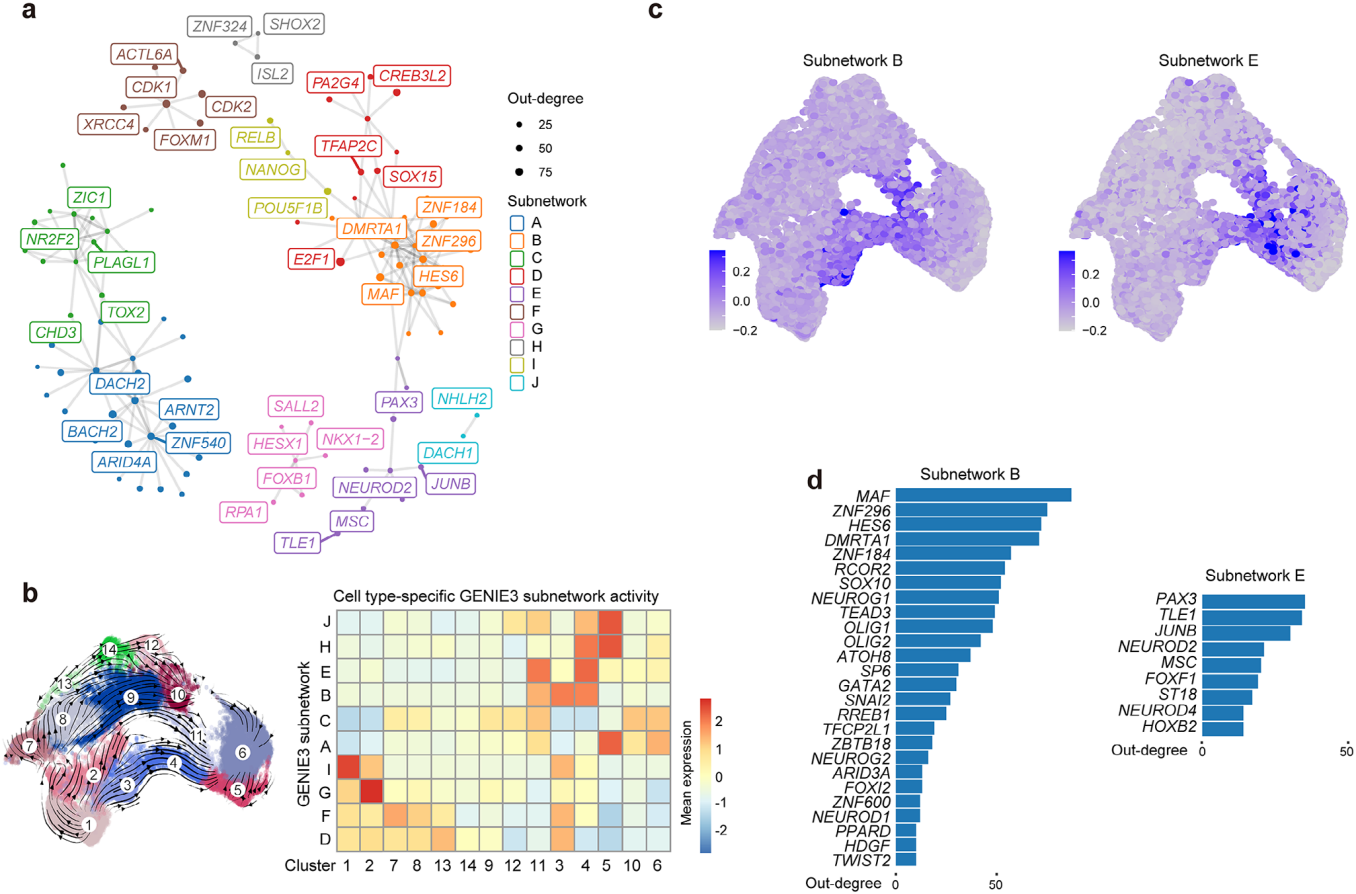
Analysis of transcriptional modules in the TF route and distinct gene regulatory networks for neurogenesis and neuronal maturation. a) GENIE3 GRN analysis showing the top 200 regulators clustered into subnetworks. Regulators were selected based on predicted regulation of the top differentially expressed genes in the entire dataset. The top five regulators in each subnetwork are labeled. b) Heatmap depicting the cell‐type specificity of GENIE3 subnetworks using the mean subnetwork activity of the scRNA atlas clusters. c) UMAP embeddings of mean scRNA expression of subnetwork‐specific genes showing two highly cell‐type‐specific subnetworks (B and E). d) Top gene ranked by out‐degree value in subnetwork B and subnetwork E.

We next aimed to infer GRNs that may play a crucial role in facilitating rapid neurogenesis and subsequent neuronal maturation. For this purpose, we analyzed scRNA‐seq data via the GENIE3 methodology in order to infer causal TF‐target networks.^[^ ^102^ ^]^ We identified 200 top regulators (ordered by the number of target genes which connected to regulators) that were clustered into ten GRN subnetworks (**Figure** **5**a); each subnetwork was associated with an scRNA‐seq cluster, indicating the significance of these drivers in orchestrating cell fate conversion within the TF route (Figure 5b,c, Figure S5, Supporting Information).

We further focused our attention on two subnetworks associated with neurogenesis and neuronal maturation stages. Subnetwork B exhibited a high correlation with cells in clusters 3 and 4, indicative of its involvement in rapid neurogenesis. On the other hand, subnetwork E showed a strong association with cells in clusters 4, 5, and 11, suggesting its role in regulating neuronal maturation (Figure 5c). Subsequently, we prioritized the identification of key TFs within subnetworks B and E by evaluating regulators based on their ability to explain coordinated variation within the data. Critical neurogenesis TFs, such as *NEUROD1, NEUROG2, HES6*, and *ZBTB18* ^[^ ^41^ ^]^ ranked high in subnetwork B, confirming the correlation of this subnetwork with neurogenesis (Figure 5d). Intriguingly, the *OLIG* family TFs, *OLIG1* and *OLIG2*, which are well‐known for their roles in oligodendrocytes and motor neuron development,^[^ ^59^, ^60^, ^61^ ^]^ exhibited high rankings within subnetwork B. Additionally, several bHLH TFs (*NEUROD2, NEUROD4*) and B‐cell related TFs (*MSC*)^[^ ^103^, ^104^ ^]^ were identified within subnetwork E, indicating novel roles in facilitating neuronal maturation (Figure 5d).

### 2.6 Perturbing Neurogenesis GRNs Causes a Developmental Delay

**Figure 6:**
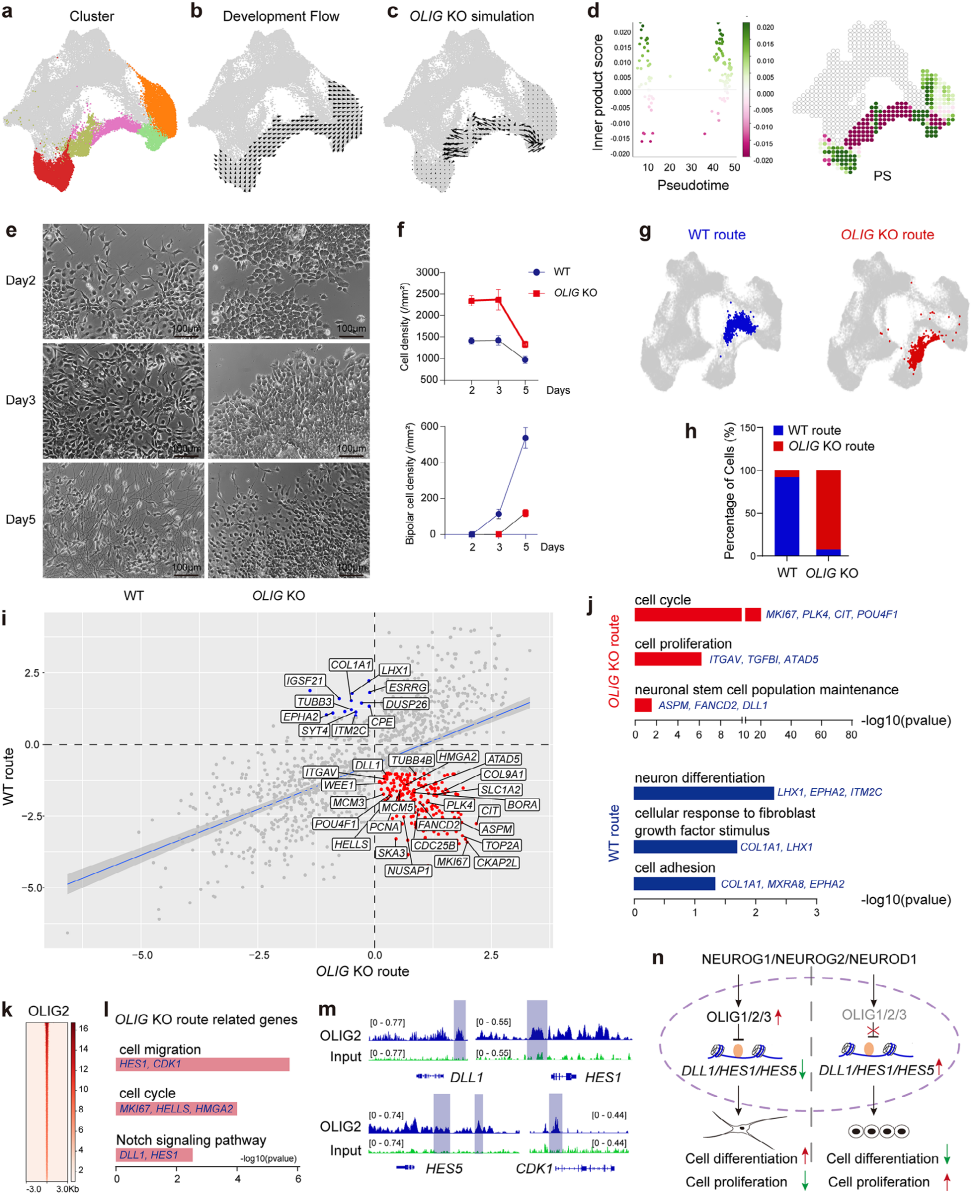
Perturbing neurogenesis GRNs and developmental delay. a) scRNA‐seq clusters that were selected for the simulation of cell‐state transitions in response to TF perturbation. b) Differentiation vectors for each cell projected onto the force‐directed graph. c) CellOracle simulation of cell‐state transition in *OLIG1/2/3* knockout (referred to *OLIG* KO) simulation. d) The inner product represents the similarity between two vectors of perturbation simulation and development flow, and the inner product of these vectors is calculated to produce a perturbation scores (PSs). e) Representative images of neuronal induction using 3N_TF OE hPSCs line (referred to WT) and *OLIG1/2/3* KO in dox‐inducible 3N_TF OE hPSCs line (referred to *OLIG* KO). f) Total cell density and bipolar cell density of WT and *OLIG* KO cells. Data are presented as mean ± SD of n = 3 independent replicates. g) Ground‐truth experimental scRNA‐seq of neuron induction using WT and *OLIG* KO cells. Neuron induction with *OLIG* KO cell line showing a distinct differentiation route (red) from WT cells (blue). h) The proportions of cells on the WT and the *OLIG* KO route. i) Scatter plot of the gene differential expression between the WT route and the *OLIG* KO route. Every point in the scatter plots shows the expression of a gene in the two conditions. We set a fold change (linear) cut‐off of ±2 and p‐value < 0,05. j) Enriched gene ontology terms for differential expressed genes between the WT route and the *OLIG* KO route. The bar graph shows the negative logarithmic p‐values. k) ChIP‐seq profile of OLIG2 within ±3 kb from the peak center (red signal) in neurons induced with 3N_TF at day 3. A total of 140373 peaks were identified using the mergePeaks function with default parameters. l) 3N_TF Enriched gene ontology terms for differentially expressed genes (from Figure 6i) that are also bound by OLIG2. m) Genome browser visualization of the OLIG2 binding tracks within specific gene loci. n) A schematic diagram illustrating the regulatory interaction of 3N_TF on OLIG1/2/3 is presented. During 3N_TF‐induced neurogenesis, 3N_TF promotes the expression of OLIG1/2/3, which in turn accelerates neurogenesis and inhibits cell proliferation by downregulating the expression of NOTCH signaling pathway‐related genes, including *DLL1, HES1*, and *HES5*. Conversely, following the knockout of OLIG1/2/3, an upregulation of *DLL1*, *HES1*, and *HES5* is observed, leading to increased cell proliferation and decreased cell differentiation.

To identify potential TFs that expedite neurogenesis, we concentrated on TFs in the subnetwork B that are specifically expressed in the TF route. The rationale for this is that while TFs expressed in both routes are likely to be fundamental to the process of neurogenesis itself, those specifically expressed in the TF route may not be critical for neurogenesis but could instead serve as key modulators of the temporal dynamics of neurogenesis. Based on this hypothesis, we focused on *OLIG* family TFs with expression restricted to the TF route and implicated as pacemakers for motor neuron generation.^[^ ^19^, ^105^ ^]^ To gain mechanistic insights, we utilized CellOracle,^[^ ^106^ ^]^ an approach that integrates single‐cell multi‐omics data to simulate changes in cell identity upon TF perturbation. Specifically, we constructed GRN models for TF‐specific clusters in the UMAP (clusters 1, 3, 4, 5, and 6) (**Figure** **6**a). The perturbation simulations, wherein *OLIG* knockout is visualized as a vector map on the 2D trajectory space, revealed a potential disruption in the neurogenesis shortcut (Figure 6b,c). Furthermore, we computed the “perturbation score” of *OLIG* knockout, and a negative score suggests that the loss of *OLIGs* leads to a delay in differentiation (Figure 6d). The cell density plot also demonstrates that simulating genetic knockouts of all three *OLIG* family members, using the inferred CellOracle GRN, results in developmental delays in neurogenesis (Figure S6a, Supporting Information).

To validate the predictions from the *OLIG* knockout simulation and ascertain the impact of *OLIG* deficiency in TF‐induced forward programming, we generated *OLIG‐1, ‐2*, and ‐*3* triple knockouts in a dox‐inducible 3N_TF OE hPSCs line (Figure S6b, Supporting Information). The knockouts were confirmed via sequencing, qPCR, and immunostaining (Figure S6b‐e, Supporting Information). Remarkably, we observed a significant delay in differentiation based on morphology compared to the wild‐type dox‐inducible 3N_TF OE hPSCs line. While the wildtype cells disaggregated and extended processes by day 3, *OLIG* knockout cells remained aggregated and proliferated as colonies, as shown by higher cell density in *OLIG* knockout cells (Figure 6e). By day 5, most wild‐type cells had matured into bipolar neurons with long neural processes, while the percentage of bipolar neurons was significantly lower in *OLIG* knockout cells (Figure 6f). However, neurogenesis ultimately proceeded in the *OLIG* knockout line, suggesting that OLIG genes are not essential for neurogenesis but rather accelerate its pace.

To further describe the differentiation trajectories of *OLIG* knockout cells, we performed time course scRNA‐seq analysis (Figure S6f, g, Supporting Information). Intriguingly, the analysis uncovered a distinct branch pathway for *OLIG* knockout cells, separate from the TF and the DS route (Figure 6g,h). Comparison of the gene expression differences between *OLIG* knockout and wide‐type cells identified 247 differentially expressed genes (absolute log2‐fold change >1) (Figure 6i). GO analyses of differentially‐expressed genes uncovered a significant upregulation of genes involved in the cell cycle, suggesting that one of the key roles of *OLIG* TFs is to facilitate the exit of cells from proliferative cycles and commit to neuronal differentiation (Figure 6j). Additionally, *OLIG* knockout resulted in the downregulation of cell adhesion molecules (e.g., COL1A1, MXRA8) typically associated with mesenchymal cells, which is reflected in the delayed loss of epithelial‐like morphology (Figure 6e,j). Furthermore, GRN analyses revealed that while regulatory nodes of neurogenesis remain stable, those associated with the cell cycle were significantly altered (Figure S6h‐j, Supporting Information). Additionally, we observed significant upregulation of *DLL1*, a component of the NOTCH signaling pathway, in *OLIG* knockout cells (Figure 6i). ChIP‐seq analyses further identified *DLL1*, along with *HES1* and *HES5*, as downstream regulatory targets of OLIG2, all of which are components of the NOTCH signaling pathway that represses neurogenesis (Figure 6k‐m).^[^ ^89^ ^]^ These results confirm that OLIG TFs may promote neurogenesis by directly modulating NOTCH signaling levels.

Collectively, these results provide evidence that during neuronal differentiation, a specific subnetwork of GRNs, centered on OLIG TFs, primarily influences the pace of neurogenesis in TF‐induced forward programming. Early in the process of in vitro differentiation, 3N_TF activates OLIG TFs, which then directly bind to *DLL1*, *HES1*, and *HES5* to repress their expression, thereby facilitating cell cycle exit and neurogenesis. Once OLIG TFs are knocked out, the expression of *DLL1*, *HES1*, and *HES5* is upregulated, causing cells to remain in a prolonged cell cycle and delaying neurogenesis (Figure 6n). This model highlights the intricate balance between proliferation and differentiation, with OLIG TFs playing a critical role in this regulatory interplay.

### 2.7 Leveraging Data on GRNs Governing Neuronal Maturation Refines TF Recipe to Expedite In Vitro Neuronal Maturation

**Figure 7:**
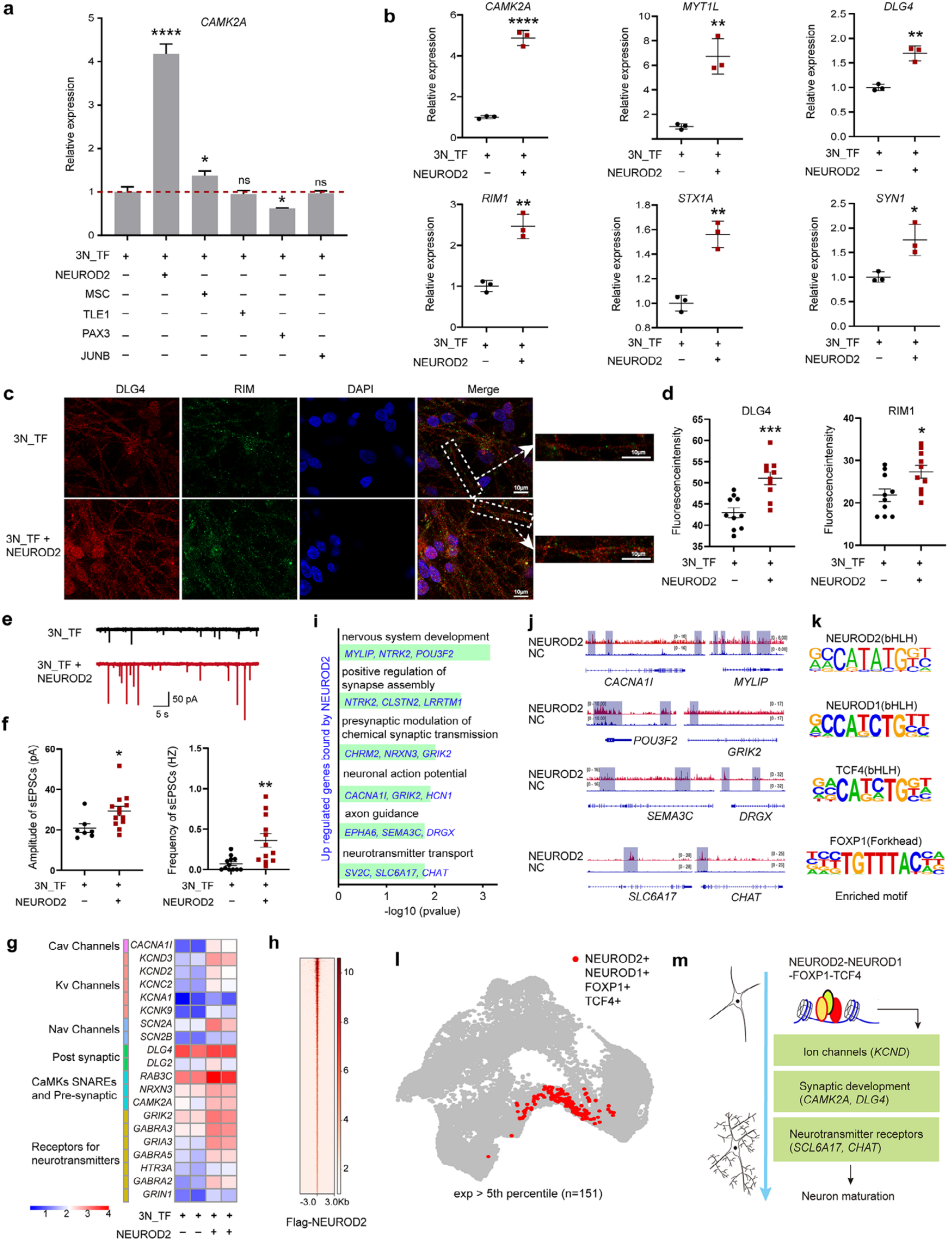
NEUROD2 promotes neuronal maturation. a) Screening of TFs for promoting neuronal maturation by RT‐qPCR. In conjunction with 3N_TF, the selected TFs were overexpressed using lentivirus, and the expression levels of *CAMK2A* were examined at day 11. Data were analyzed from three technical replicates. hPSCs‐female cell line was used in this experiment. *p < 0.05; **p < 0.01; ***p < 0.001; ****p < 0.0001. b) The expression levels of genes related to neuronal maturation at differentiation day 11. Data represent the mean ± SD of n = 3 independent replicates. Two hPSCs cell lines (hPSCs‐female and hPSCs‐male) were used for this experiment. *p < 0.05; **p < 0.01; ***p < 0.001; ****p < 0.0001. c) Representative immunofluorescence images for DLG4 and RIM in 3N_TFs induced neurons at day27. Scale bars are 10 µm. d) Quantification of the fluorescence intensity of DLG4 and RIM staining. Data are presented as mean ± SD of n = 3 independent replicates. *p < 0.05; **p < 0.01; ***p < 0.001; ****p < 0.0001. **e**) Representative voltage clamp recordings of spontaneous excitatory postsynaptic currents (sEPSCs) from 3N_TF induced neurons at day 25 for each condition. f) The quantification of the frequency and amplitude of sEPSCs. Data represent the mean ± SD of n = 3 independent replicates. *p < 0.05; **p < 0.01; ***p < 0.001; ****p < 0.0001. g) Heatmap illustrating the expression of genes related to neuronal excitability and connectivity 3N_TF at day 11. h) ChIP‐seq profile of flag‐NEUROD2 within ±3 kb from the peak center (red signal) in neurons induced with 3N_TF and flag‐NEUROD2 at day 10. A total of 2941 peaks were identified using the macs2 with default parameters. i) 3N_TF Enriched gene ontology terms for upregulated genes that were bound by NEUROD2. j) Top five enriched motifs identified by ChIP‐seq analysis. k) Genome browser visualization of the NEUROD2 binding tracks within specific genomic loci. l) UMAPs showing the existence of NEUROD1, NEUROD2, FOXP1, and TCF4 co‐expression cells. m) The schematic diagram of NEUROD2 promoting neuronal maturation. NEUROD2 forms a regulatory complex with NEUROD1, TCF4, and FOXP1 to activate genes associated with ion channels (*KCND* family), synaptic development (*CAMK2A, DLG4*), and neurotransmitter receptors (*SLC6A17, CHAT*) to accelerate in vitro neuronal maturation.

Observations indicated that despite differences in the timing of neurogenesis between the TF and DS routes, the speed of neuronal maturation is not a continuous variable but instead remains consistent. Motivated by the notion that manipulating TFs can not only direct cell fate but also modulate differentiation timing, we sought to expedite neuronal maturation in vitro via modulation of the TFs identified in subnetwork E, which is enriched at post‐neuronal fate commitment, where pro‐maturation genes are likely to be activated. By administering either synthetic mRNA encoding these TFs or a lentiviral overexpression system, we overexpressed top five ranked TFs of subnetwork E and evaluated their impact on neuronal maturation by the assessment of the expression of *CAMK2A*, a key marker gene involved in this process.^[^ ^33^, ^107^ ^]^ Among the five TFs tested, the overexpression of NEUROD2, in combination with 3N_TF, markedly upregulated *CAMK2A* expression, with *MSC* overexpression exhibiting a less pronounced effect (**Figure** **7**a).

Further analysis revealed that overexpression of NEUROD2 or MSC significantly enhanced the expression of a core set of maturation‐related genes, including *CAMK2A*, *MYT1L*, *DLG4*, *RIM*, *STX1A*, and *SYN1* ^[^ ^17^, ^31^, ^108^ ^]^ at day 10‐15, compared to the control (Figure 7b, Figure S7a,b, Supporting Information). Further, by day 25‐30, cells overexpressing NEUROD2 or MSC exhibited a greater number of synapses, as indicated by the staining of the presynaptic and postsynaptic markers RIM and DLG4 (Figure 7c,d, Figure S7c,d, Supporting Information). Electrophysiological characterization at day 25 also demonstrated that while control cells had sparse or low‐amplitude spontaneous excitatory postsynaptic currents (sEPSCs), those overexpressing NEUROD2 or MSC displayed repetitive sEPSCs with heightened amplitude and accelerated kinetics, closely resembling the electrophysiological features of control cells at day 38 (Figure 7e,f, Figure S7e,f, Supporting Information). These findings indicate that NEUROD2 or MSC overexpression accelerates neuronal maturation without inducing abnormal electrophysiological features, maintaining functional integrity consistent with normal cortical neuron development. Notably, NEUROD2 and MSC overexpression also accelerated neuronal maturation in DS‐induced neuronal differentiation, as evidenced by elevated mRNA and protein expression of mature neuronal markers (Figure S7k‐p, Supporting Information). Thus, NEUROD2 and MSC act as conserved temporal regulators of neuronal maturation across both TF‐induced and DS‐induced routes.

To explore the regulatory mechanisms behind this accelerated maturation, we examined the genomic binding of NEUROD2, and gene expression profiles post‐overexpression. Strikingly, NEUROD2 was found to bind to genes associated with ion channels, synaptic development, and neurotransmitter receptors, likely enhancing their expression through direct transcription activation (Figure 7g–j). Similar results were also obtained for MSC overexpression (Figure S7g–j, Supporting Information). Motif analyses revealed consensus motifs for NEUROD1, TCF4, and FOXP1, which known to promote neuronal maturation,^[^ ^109^, ^110^, ^111^, ^112^ ^]^ alongside NEUROD2 (Figure 7k). These TFs are co‐expressed with NEUROD2 in mature neurons (Figure 7l), suggesting that NEUROD2 may form complexes with these TFs to synergistically activate the neuronal maturation program (Figure 7m).

In conclusion, these findings highlight the importance of specific TFs, particularly NEUROD2 and MSC, in modulating neuronal maturation during in vitro differentiation. Specifically, ectopic NEUROD2 forms a regulatory complex with NEUROD1, TCF4, and FOXP1 to activate genes associated with ion channels (*KCND* family), synaptic development (*CAMK2A*, *DLG4*), and neurotransmitter receptors (*SCL6A17*, *CHAT*) to accelerate in vitro neuronal maturation (Figure 7m). With this knowledge, an improved TF recipe that markedly accelerates neuronal maturation and enables the production of neurons with mature marker genes, functional synapses, and postsynaptic activity within a 30‐day differentiation period has been developed.

## 3 Discussion

TFs are master regulators of cell fate determination and can be used to convert cells from one fate to another. In this study, we address the hypothesis that TFs that are not essential for neurogenesis may nonetheless expedite neurogenesis relative to the DS route, thought to mimic neurogenesis in vivo. We examined the *OLIG* family of TFs, members of which are exclusively expressed in the TF route of neurogenesis, and identified potential pace setters in motor neuron generation.^[^ ^19^ ^]^ Although their expression and function has not been reported in embryonic forebrain development, they have been detected during glutamatergic neuron differentiation in vitro^[^ ^38^, ^113^ ^]^ and in forebrain organoid formation.^[^ ^114^ ^]^ However, their roles in these in vitro differentiation systems remain unexplored. Our study demonstrates that, in contrast to their roles in motor neuron development, the absence of OLIG TFs did not impede neurogenesis; rather, the absence induced a differentiation delay. Mechanistic insights reveal 3N_TF‐OLIG‐NOTCH regulatory axis that controls the pace of neurogenesis. Specifically, 3N_TF activates OLIG TF, which then directly binds to and regulates cell cycle genes and NOTCH signaling genes to facilitate cell cycle exit and commitment to neuronal differentiation. Interestingly, we note that the expression of OLIG is restricted in cells that undergo neurogenesis in the TF route, and its expression is immediately silenced once neurogenesis completes. It seems like OLIG is specifically expressed to ensure the fast neurogenesis in TF‐induced forward programming and silenced at the exact timing to ensure a dedicated cell fate commitment towards glutamatergic neurons, as prolonged expression after neurogenesis would confuse cells for motor neuron or oligodendrocyte differentiation. The mechanism of controlling the timing of OLIG TF expression in TF‐induced forward programming is of great interest to understand cell‐intrinsic regulation of cell fate determination and timing control. Also, we observed the activation of *DLL3* and *HES6*, which also regulate NOTCH signaling and promote neurogenesis signals.^[^ ^50^, ^52^ ^]^ The activation of their expression may explain why the ablation of OLIG TFs did not significantly hinder neurogenesis compared to the DS route. Further investigations into the potential redundant or cooperative roles of these genes in regulating the pace of neurogenesis would be valuable.

Leveraging results observed in these studies, we forced TF expression in neuronal maturation GRNs to accelerate in vitro neuronal maturation. Our results identify NEUROD2 as a critical modulator of neuronal maturation during in vitro differentiation in both TF‐ and DS‐induced systems. Interestingly, we had initially excluded NEUROD2 from our list of TFs aimed at promoting rapid and efficient neurogenesis. However, upon screening for TFs that expedite neuronal maturation, NEUROD2 emerged as a standout candidate. This indicates that while TFs may be essential for the same differentiation pathway, they can function at different times to fulfill specific roles. Importantly, NEUROD2 is indicated as a critical TF involved in both neurogenesis and neuronal maturation in embryonic brain development.^[^ ^115^, ^116^, ^117^ ^]^ We also analyzed a developing human cortex dataset and confirmed its upregulation in maturing cortical neurons.^[^ ^64^ ^]^ Our findings, along with published studies on NeuroD2 in mice, highlight the importance of NEUROD2 as a conserved temporal regulator in neuronal maturation across species and contexts.^[^ ^115^, ^116^, ^117^ ^]^

Together our findings combined with published studies elucidate a hierarchy within GRN networks, positioning neuron‐essential TFs at the apex to induce a robust cell fate commitment. While loss of neuron‐essential TF disrupts neurogenesis, altering key nodes of downstream GRNs does not abolish or inhibit neurogenesis; rather, alterations of downstream nodes act as a rheostat to finely tune the differentiation trajectories towards the ultimate neuronal fate and thus lead to variations in developmental timing. Accelerating in vitro neuronal maturation holds significant promise for establishing more physiologically relevant and disease‐related cellular models that faithfully recapitulate the characteristics of mature neurons.

Considering that overall gene expression profiles can be classified into distinct expression modules, each TF can be viewed as a molecular “switch” within the regulatory network. When activated, these TF switches induce, or turn on, the expression of specific modules. The influence of TFs on cell fate decisions is determined by their capacity to activate modules that drive cell fate conversion. Pioneer TFs of cell fate, whose combined effects are potent enough to activate all necessary modules for programming cell fate, are of particular interest. In this study, we uncovered evidence for at least four gene modules that must be directly activated to facilitate the establishment of new cellular programs. The first module consists of chromatin remodeling TFs that initiate the reorganization of the chromatin landscape, thereby facilitating the binding of other factors. The second module includes genes involved in reshaping the cytoskeleton and extracellular matrix. Cell shape plays a pivotal role in coordinating cell fate determination, and alterations in cytoskeletal structures or cellular movement can modulate signaling pathways that lead to gene expression changes influencing cell fate determination. The third module encompasses genes related to mRNA splicing, which ultimately regulate cell fate decisions. During differentiation, hundreds of genes in human pluripotent stem cells undergo isoform changes. Particularly in the developing cerebral cortex, cell type‐specific alternative splicing governs cell fate determination, and disruptions to this process can lead to brain malformations.^[^ ^94^, ^118^ ^]^ The fourth and last module significantly activated by pioneer TFs comprises lineage‐specific TFs, which coordinate gene expression and reinforcement towards the ultimate cell fate at various molecular levels. In our dataset, proneuronal TFs like *NEUROG3*, *ATOH*, *HES6*, and *ZBTB18* ^[^ ^41^ ^]^ are directly activated by proneuronal TFs to promote neurogenesis. These four modules have been indicated by previous studies as essential modulators for cell fate conversion.^[^ ^94^, ^118^, ^119^, ^120^, ^121^ ^]^ Further investigation of the necessity and sufficiency of these four modules may have extensive implications for the rational selection of pioneer TFs for programming different lineages from hPSCs.

After closer inspection of GRNs, we investigated the impact of a trio of proneuronal TFs on the establishment of glutamatergic neuronal fate from a pluripotent state. While NEUROG2 can promote diverse neuronal subtypes,^[34,^ ^35^, ^99^ ^]^ our findings show that the synergistic expression of NEUROG1, NEUROD1, and NEUROG2 results in a less heterogeneous population of endpoint neurons, predominantly of the glutamatergic lineage. Notably, we and others observed that increasing the dosage and duration of exogenous TF exposure significantly reduced heterogeneity.^[^ ^35^ ^]^ Mechanistic analyses also demonstrated that co‐expression of bHLH TF partners with NEUROG2 results in reorganization of the genomic binding landscape of NEUROG2.^[^ ^40^ ^]^ This coordinated expression of bHLH TF partners with NEUROG2 not only specifies glutamatergic neurons but also silences non‐glutamatergic programs, in alignment with recent reports, albeit with different partner TFs.^[^ ^40^ ^]^ Moreover, culture conditions or signaling molecules can also redirect NEUROG2‐expressing cells into different neuronal subtypes.^[^ ^99^ ^]^ Collectively, these observations suggest that pioneer TFs, such as NEUROG2, occupy the pinnacle of the transcriptional regulation hierarchy, with the flexibility of subtype cell fate determined by other TFs or signaling molecules.

TF‐induced forward programming and stepwise cellular differentiation of neurons from hPSCs are widely employed tools for studying human neuronal development and disease.^[^ ^122^, ^123^, ^124^ ^]^ However, the differences between these methods are often overlooked. The data provided here present a comprehensive molecular roadmap of in vitro human neuronal differentiation at the single‐cell level, uncovering distinct trajectories and intermediate cellular states inherent to TF‐ and DS‐induced differentiation methods. The detailed transcriptomic analyses unveiled two distinct acceleration steps in the TF route: the initial commitment to neuronal fate, bypassing the NE stage, followed by direct neurogenesis from RG cells, which skips IPs. In contrast, the DS route reflects an indirect neurogenesis process that involves IPs. The distinction in neurogenesis pathways primarily arises from differential expression of NOTCH signaling, where neurogenesis in the DS route is prolonged due to mutual inhibition of NOTCH signals and proneuronal genes, while the TF route facilitates rapid neurogenesis through the activation of proneuronal genes. The regulation of timing may involve distinct subnetworks of TFs: DS differentiation may be controlled by subnetwork C, which includes TFs like *NR2F2* and *PAX6*, both of which have been shown in prior studies to play critical roles in regulating the timing of neuronal differentiation.^[^ ^125^, ^126^, ^127^ ^]^ In contrast, the TF route is dominated by *OLIG1, OLIG2*, and *OLIG3* (Figure 1j, Figure S5, Supporting Information). Recent work underscores the importance of direct and indirect neurogenesis pathways in understanding the cellular and molecular mechanisms underlying the generation of distinct neuronal subtypes with varying functions in the human cortex.^[^ ^10^, ^11^ ^]^ Collectively, by elucidating the role of TF combinations in directing neuronal fate, this study contributes to the understanding of transcriptional mechanisms that govern human cell fate determination and temporal control during neurogenesis. This knowledge may be harnessed to develop targeted differentiation protocols, facilitating advancements in the field of neural regeneration and providing new avenues for studying and treating neurological disorders.

## 4 Experimental Section

### Cell Lines

The human hPSCs lines were purchased from CELLAPY company (derived from skin fibroblasts, Cellapy, Beijing, China). The cell line was authenticated by immunostaining for cell‐type specific proteins and tested as negative for mycoplasma contamination. The human hPSCs lines were maintained on feeder‐free Matrigel‐coated plates as previously described.^[^ ^128^ ^]^ All cultures were grown at 37 °C with 5% CO2 in mTeSR1 medium and were passaged using Collagenase type IV (Technologies) as aggregates or Accutase (STEMCELL Technologies) as single cell. 293T cells were maintained in high‐glucose DMEM with 10% fetal bovine serum, and 1% penicillin streptomycin and were passaged using TrypLE (Gibco).

### Generation of In Vitro Transcription (IVT) Templates

The ORF DNA sequences were synthesized by Integrated DNA Technologies (NEUROG1, NEUROG2, NEUROG3, NEUROD1, NEUROD2 and ASCL1) or created from cDNA (MSC). The ORFs were then cloned into pCR2‐UTR‐R1R2 vector.^[^ ^97^ ^]^ pCR2‐UTR‐R1R2 plasmids containing ORFs were linearized by XhoI (New England Biolabs) and purified. 1ng of linearized plasmids were used as PCR template and PCR reactions were performed with HiFi Hotstart (KAPA Biosystems, Woburn, MA) per the manufacturer's instructions. PCR products were purified with DNA clear and concentrator spin columns (Zymo Reseaerch) and adjusted to 100ng/ul concentration before in vitro transcription.

### Generation of mod‐mRNAs

RNA was synthesized with the MEGAscript T7 kit (Ambion, Austin, TX), with 1.6 ug of purified tail PCR product to template each 40 uL reaction. Co‐Capping in vitro transcription was performed with 6mM final concentration of 3′‐0‐Me‐m7G(5′)ppp(5′)G ARCA cap analog (New England Biolabs), adenosine triphosphate and guanosine triphosphate, 5‐methylcytidine triphosphate and pseudouridine triphosphate (TriLink Biotechnologies, San Diego, CA). Reactions were incubated 6 hr at 37 °C and DNase treated as directed by the manufacturer. RNA was purified with MEGAclear spin columns (Thermo Fisher), then treated with Antarctic Phosphatase (New England Biolabs) for 30 min at 37 °C to remove residual 5′‐triphosphates. Treated RNA was purified again with MEGAclear kit, quantitated by Nanodrop (Thermo Scientific, Waltham, MA), and adjusted to 100 ng/mL working concentration by addition of Tris‐EDTA (pH 7.0). The mRNA products were quality‐controlled by running 1 ul mRNA on tapestation 2200 (Agilent).

### RNA Transfection

Transcription factor mRNAs were pooled together at 250 ng per factor per well of a 24 well plate. Pluripotent stem cells were digested with accutase to obtain a single‐cell solution and resuspended in mTeSR1 supplemented with 10 uM Y27632. Cells were seeded at a density of 2.5 × 10^4^ cells/cm^2^ onto Matrigel (Corning)‐coated plates, and the mRNA mixture was transfected using Lipofectamine Stem reagent (Thermo Fisher) according to the manufacturer's instructions. Briefly, mRNA cocktail in 25 ul OptiMEM was mixed with 4 ul LipoStem in 25 ul OPTI‐MEM and incubated at room temperature for 10 minutes for complex formation. Complexes were then added dropwise to wells.

### Lentivirus Production and Transduction

293T cells were seeded in T75 flasks and were transfected the next day at ∼90% confluency. For each T75 flask, 12 µg of plasmid containing the vector of interest (NEUROD2, MSC, TLE1, PAX3, JUNB), 9 µg of psPAX2, and 3 µg of pMD2.G were transfected using 62 µl of PEI and media was changed 6h later. Virus supernatant was harvested 48h post‐transfection, filtered with a Centrifugal Filters (Millipore, UFC910096), aliquoted and stored at ‐80 °C. For transduction, hPSCs were seeded in cell culture dishes and transduced with 5 µL of lentivirus per 1 × 10^5^ cells (normalized to a titer of 1 × 10^8^ TU/mL, MOI = 5) in the presence of 0.5 µg/mL puromycin. After 48h, media was refreshed with neural induction media for neural differentiation.

### Differentiation Protocol for 3N_TFs Induced Neurogenesis

At day 0, hPSCs were disassociated with Accutase and plated onto Matrigel coated plate at the density of 50000 cells/cm^2^ in mTeSR1 media with Y27632 (10uM, TargetMol, T1725). At Day 1, cells were transfected with mRNA (3N_TF) and incubated for 3 hours. After 3 hours, media was changed to fresh mTeSR1 with Y27632 for 2 hours recovery. Repeat this transfection 3 times in 1.5 days. After the last transfection, change cell culture media to StemFit (StemFit, AK02N), NSC, NPC, Neuron or NIM2i (Neural Induction Media) (STEMCELL, 0 5835) supplemented with 1% Penicillin‐Streptomycin. The formulation for NSC culture medium is composed of KnockOut DMEM/F‐12 (Gibco, 12 660 012), GlutaMAX Supplement (2 mM, Gibco, 35 050 061), Basic Fibroblast Growth Factor (bFGF) (20 ng/mL, Gibco, 13256‐029), Epidermal Growth Factor (EGF) (20 ng/mL, Gibco, AF‐100‐15), and StemPro Neural Supplement (2%, Gibco, A1050801). For NPC culture medium, the composition includes DMEM/F‐12 (Gibco, 11 320 033), STEMdiff Neural Progenitor Medium Supplement (STEMCELL, 0 5833), L‐glutamine (2 mM, Gibco, 25 030 081). The Neuron media formulation consists of Neurobasal‐A Medium (Gibco, 10 888 022), B27 (2%, Gibco, 12 587 010), GlutaMAX (1%, Gibco, 35 050 061), 2‐Mercaptoethanol (50 mM, Gibco, 21 985 023), Brain‐Derived Neurotrophic Factor (20 ng/mL, BDNF, CELLAPY, CA32006), GDNF (20 ng/mL, Gibco, 450‐10), Cyclic AMP (0.1 mM, cAMP, Sigma, D0627), and Ascorbic Acid (0.2 mM). The formulation of NIM2i consists of Neural Induction Media supplemented with two Smad inhibitors LDN193189 (250 nM, TargetMol, T1935) and SB431542 (10 µM, TargetMol, T1726). At day4, change cell culture media to Neural Basal Media (Gibco, 21 103 049) supplemented with B27 (2%), BDNF (20 ng/ml) and cAMP (0.1 mM).

### Neuronal Differentiation with dox‐inducible 3N_TF OE Cell Line

At day 0, cells were disassociated with Accutase and plated onto Matrigel coated plate at the density of 50000 cells/cm^2^ in mTeSR1 media with Y27632 (10 uM). At Day 1, change cell culture media to mTeSR1 supplemented with dox (doxycycline hyclate) (2 ug/ml, T1687L, TargetMol) and Y27632 (10 uM). At day2, change cell culture media to mTeSR1 supplemented with dox. At day2, change cell culture media to NIM2i supplemented with dox. At day4, change cell culture media to Neural Basal Media supplemented with N2 (1%, Gibco,17 502 048), B27 (2%), BDNF (20 ng/ml) and cAMP (0.1 mM).

### DS Differentiation Protocol

As described before,^[^ ^30^ ^]^ at day 0, hPSCs were disassociated with Accutase and plated onto Matrigel coated plate at the density of 250000 cells/cm2 in NIM2i and Y27632 (10uM). From day 1 to day 2, change cell culture media to NIM2i supplement with XAV939 (2 µM, TargetMol, T1878). From day 3 to day 4, change cell culture media to NIM2i supplement with XAV939 (1 µM), PD0325901 (0.4 µM, TargetMol, T6189), SU5402 (2 µM, TargetMol, T6996) and DAPT (5 µM, TargetMol, T6202). N2B27 medium was added to NIM2i at 1/3 (v/v) from day5, with 1/3 increment every other day. From day 5 to day 6, inhibitors PD0325901 (0.4 µM), SU5402 (2 µM), DAPT (5 µM) were added to the medium of NIM2i/N2B27 (2:1). From day 7 to day 8, PD0325901, SU5402, DAPT were added to NIM2i/N2B27 (1:2). At day 9, 100% N2B27 media supplement with BDNF and cAMP was used. An outline of DS neuron differentiation scheme is presented in Figure 1e.

### Long‐term Live Cell Imaging of Neuronal Differentiation

After overexpression of 3N_TF in hPSCs, live cells were imaged for over about 50 hours with Nanolive’ s 3D Cell Explorer‐fluo.

### Quantitative PCR

Total RNA for qPCR was extracted from cells using a RNeasy Plus Mini kit (Qiagen) and RNA (1 µg) was reverse transcribed using the HiScript III RT SuperMix system. qPCR was performed using SYBR Green PCR Master Mix (from vazyme) with the data normalized to housekeeping genes HSP or GAPDH. The primer sequences are listed in Supplementary Table S1.

### Western Blot

Protein was extracted with RIPA lysis buffer containing protease inhibitor cocktail. Concentrations were measured BCA reagent (Beyotime Biotechnology, Shanghai, China). For Western blot analysis, 30 µg of protein per sample was loaded onto SDS‐polyacrylamide electrophoresis gels, transferred to PVDF membranes (Roche, Switzerland), and probed with appropriate primary and HRP‐conjugated secondary antibodies (CST). The blots were visualized using ImmobilonTM Western Chemiluminescent HRP Substrate.

### Immunofluorescence

Cells were fixed with 4% paraformaldehyde at room temperature for 20 min, permeabilized with 0.3% Triton X‐100 in PBS (PBST) for 10 min and blocked for 45 min at room temperature in PBST containing 5% normal donkey serum (Jackson ImmunoResearch Laboratories). Primary antibodies were diluted at 1:200 in blocking solution and incubated at 4 °C overnight. The following primary antibodies were used in this study: SATB2 (ABclonal, Cat. A19837), TBR1 (proteintech, Cat. 17490‐1‐AP), VGLUT2 (Affinity, DF13296), MYT1L (Abcam, Cat. ab229494), RIM (Synaptic Systems, Cat. 140 213), DLG4 (neuromab, Cat. 75‐028), EOMES (CST, D8D1R), MAP2 (proteintech, Cat. 17490‐1‐AP), TUBB3 (BioLegend, Cat. 801 201), OLIG2 (Sigama, AB9610), OLIG3 (Abcam, ab129297), CDH1 (CST, 24E10), CDH2 (CST, D4R1H). Appropriate Alexa Fluor 488, 594 or 647‐conjugated secondary antibodies (Jackson ImmunoResearch Laboratories) were diluted at 1: 300 in PBS containing 0.1% bovine serum albumin (BSA) and incubated at room temperature for 1 h. For nuclear staining, 4,6‐diamidino‐2‐phenylindole (1 µg/ml, DAPI) was used. Images shown are representative of at least three independent experiments. Immunofluorescence images were taken with a W1 spinning disc confocal microscope (Ti2E, NIKON) with a 100X oil objective or Echo Revolve microscope (Echo Laboratories, San Diego, CA, USA) and analyzed using Fiji ImageJ.

### Electrophysiology

Electrophysiological recordings were conducted without astrocytes co‐culture, as previously described with slight modifications.^[^ ^129^ ^]^ Shortly, coverslips with differentiated neurons were maintained in artificial cerebrospinal fluid (ACSF) extracellular solution containing NaCl (125 mM), KCl (2.5 mM), NaH2PO4 (1.25 mM), NaHCO3 (25 mM), D‐Glucose (2.5 mM), Sucrose (22.5 mM), CaCl2 (2.5 mM), MgCl2 (1.2 mM), pH 7.3–7.4. The ACSF was bubbled with 95% O2 and 5% CO2 during recording. Patch pipettes were pulled (3‐5 MΩ tip resistance) with a PC‐100 micropipette puller (Narishige) filled with internal pipette solution contained (in mM) K‐Gluconate (126 mM), HEPES (10 mM), EGTA (0.05 mM), KCl (4 mM), Mg‐ATP (4 mM), Na4‐GTP (0.3 mM), Phosphocreatine (10 mM), the pH was adjusted to 7.2 with KOH. Cell membrane potentials were held typically at −70 mV and depolarized to test potentials from −100 to +80 mV in 10 mV increments to record the sodium and potassium currents. For current‐clamp recordings, a hyperpolarized current was injected into neurons to membrane potentials around −70 mV. Action potentials were elicited by step‐depolarized currents from ‐100 to +80pA. The cells were held at 0 pA to record the resting membrane potentials. For synaptic functional analysis, the internal solution contained CsCl (40 mM), K‐Gluconate (90 mM), HEPES (10 mM), EGTA (0.05 mM), NaCl (1.8 mM), KCl (3.5 mM), MgCl2 (1.7 mM), Mg‐ATP (2 mM), Na4‐GTP (0.4 mM), Phosphocreatine (10 mM) and additional QX‐314 (5 mM) for evoked ePSCs. EPSCs were pharmacologically isolated by adding PTX (100 µM) into the ACSF. For evoked EPSCs recording, stimuli were delivered to the neuron through a concentric bipolar electrode (Cat. CBBEB75, FHC) with an isolated pulse stimulator (Model 2100, A‐M Systems). Whole‐cell patch‐clamp recordings were performed using an Axon 700B amplifier. Data were filtered at 2 kHz, digitized at 10 kHz and collected using Clampex 10.2 (Molecular Devices). Series resistance was compensated to 60–70%, and recordings with series resistances of >20 MΩ were rejected. The data were analyzed using Clampfit 10.2.

### Single‐cell RNA‐seq and Data Analysis (Dissociation of Neuron and Single‐cell RNA‐seq)

Cells were disassociated with Accutase to single cell and then scRNA‐seq libraries were prepared using DNBelab C Series Sinlge‐Cell Library Prep set (MGI, 1000021082)^[^ ^130^ ^]^ or 10X Genomic Chromium Single Cell library kit.^[^ ^131^ ^]^ For MGI process (hPSCs‐female cell line), followed by the Library Prep kit (droplet encapsulation, emulsion breakage, mRNA captured bead collection, reverse transcription, cDNA amplification and purification), the single cell suspensions finally were converted to barcoded scRNA‐seq libraries. Libraries were sequenced using a MGISEQ‐2000 sequencer, achieving an average sequencing depth of 19171 reads per cell, with an average of 4698 genes identified per cell. For the 10X process (hPSCs‐male cell line), scRNA‐seq libraries were prepared using the Chromium Single Cell 3′ Library & Gel Bead Kit according to the manufacturer's instructions, as described previously,^[^ ^131^ ^]^ and sequenced on the Illumina NovaSeq instrument, achieving an average sequencing depth of 5733 reads per cell, with an average of 2594 genes identified per cell.

### scRNA‐data Processing of all DS and TF Samples (MGI Process)

scRNA‐data sequenced by MGI process (hPSCs female cell line) of all DS and TF samples were processed by DNBelab C Series scRNA analysis software (https://github.com/MGI‐tech‐bioinformatics/DNBelab_C_Series_scRNA‐analysis‐software), and DNBelab C Series HT scRNA analysis software (https://github.com/MGI‐tech‐bioinformatics/DNBelab_C_Series_HT_scRNA‐analysis‐software) with default parameters and hg38 human genome to generate the feature‐cell count matrix for each samples. All these samples were then performed quality control, integration, and analysis processes by Seurat V4.4.0. In brief, cells with greater than 200 detected genes and mitochondrial gene expression percentages fewer than 20 were kept as the first step of quality control process. After this quality control process, R package DoubletFinder and manual selections were used to remove potential doublet cells and fragment cell contaminations.

### scRNA‐data Processing of all DS and TF Samples (10× Process)

scRNA‐data sequenced by 10X process (hPSCs male cell line) of 4 samples were processed by Cell Ranger 8.0.1 with default parameters and GRCh38 human reference genome to generate the feature‐cell count matrix. All these samples were then performed quality control, integration, and analysis processes by SeuratV4.4.0. In brief, cells with greater than 100 detected genes and less than 20% total mitochondrial gene expression were retained for analysis. After this quality control process, R package DoubletFinder was used to remove potential doublet cells with doublet rate parameter set as 40%. We compute the Pearson correlation using cor() between the expression profile of each cell (hPSCs male samples sequenced by 10X) and that of each cell in Figure 1f reference (hPSCs female samples sequenced by MGI). The calculation only used the top 2000 high variable genes (HVGs) identified by function FindVariableFeatures() in Seurat package. The UMAP embedding of each query cell was assigned with that of the most related reference cell correspondingly. Finally, we got the UMAP projection of cells derived from hPSCs male cell line according to the relationship between two datasets.

### Cell Accommodation and Composition

In order to accommodate differences in cell composition in each method in their analyses, the count matrix with QC and doublet removed was then performed into the accommodation process, i.e., the cell integration (batch correction) process. FastMNN was employed as the integration algorithm for sample integration based on cellular gene expression profiles. First, the function fastMMN from the R package SeuratWappers V1.1.2 was used with default parameters. Then, clustering was performed using R package Seurat V4.4.0 with the resolution parameter set to 0.4, resulting in 14 clusters. After this process, a total of 22 947 cells and 45 166 genes count matrix were retained which contained 7 DS samples and 12 3N_TF samples (3N_TF day2‐day5 with all 3N_TF expressions). Furthermore, all retained TF and DS samples were normalized with NormalizeData() with all default parameters before the integration process, and then been scaled by the function ScaleData() after the integration process, respectively. After the scaling process, the function RunUMAP was excuted with all default parameters on top 20 FastMNN reduction componements. This combined seurat object was then executed the 2‐demesion trajectory map with R package monocle3^[^ ^132^ ^]^ (V1.3.4) with default parameters. In this step, we defined the cell composition of TF and DS routes by the function choose_graph_segments(). The velocity info and visualizations are obtained by software scvelo V0.3.1^[^ ^133^ ^]^ respectively.

### Cell annotation

With unsupervised clustering, 14 distinct clusters were identified based on cellular gene expression profiles. Once clusters are identified, the function FindAllMarkers() (Seurat v4.4.0) was used to identified the top100 markers for each cluster and then mutually annotated cell types with known cell type markers (cell type markers are displayed in Table S2, Supporting Information). We assigned cell type labels to each cluster by comparing known marker gene expression levels. For clusters sharing the same marker gene (e.g., radial glial), we further refined the annotations based on additional characteristic genes. For example, radial glial with high TOP2A expression were classified as dividing radial glial. While some clusters shared certain marker genes, they had distinct gene expression profiles and the annotation aligns closely with their unique gene expression features.

### Monocle 3 for Cell Trajectory Analysis Based on Gene Expression Dynamics

In order to better understanding the cell differentiation map, the Monocle 3 was induced as a trajectory algorithm of both TF and DS cells to present how the gene expression dynamics of different cell types affect TF‐driven generation of neurons by using reversed graph embedding (RGE), especially some complexed cell growth trajectories in those TF‐driven generation of glutamatergic neurons, i.e., showing how the same neuronal types being made in each case by different differentiation trajectories. First, we processed the preprocessing and dimension reduction by default parameters, and then conducted the embedding process with function Embedding() by “umap” parameter. Furthermore, we conducted the order cell process by all default parameters, and then manually choose the root node by the function choose_graph_segements().

### “De‐novo” Gene Regulation Network (GRN) Construction of DS and TF Samples

All DS and TF scRNA samples were chosen to perform GRN construction that refined by scRNA data only (i.e., the “De novo” GRN). In brief, integrated data of TF and DS were aggregated into 483 metacells and 18 959 genes via R package metacell 0.3.7, then passed to SCENIC and cNMF. In this step, genes were also filtered via the threshold of a minimum overall count of 10 for all metacells, and at least 2 in each metacell. Moreover, those genes are also required to be presented in RcisTarget gene databases hg38__refseqr80__500 bp_up_and_100 bp_down_tss.mc9nr (hg38, 500 bp upstream of TSS, and 100 bp downstream) and hg38__refseq‐r80__10 kb_up_and_down_tss.mc9nr (hg38,10kb up and down of TSS) genes. The cNMF is used to calculate top genes and gene spectra score with a optimal k = 22, and SCENIC is used to calculate the raw GRN via method genie3. The final GRN is selected from the raw GRN by retaining the connection weight > 0.01, and the target genes which were overlapped with cNMF programming results was retained in order to remove potential noise. The top 200 regulators were retained and clustered via R package leiden (V0.4.3.1) with a resolution parameter of 0.1 and been visualized via R package igraph (V1.5.1) and ggplot2 (V3.4.4).

### ATAC‐seq Based Gene Regulation Network (GRN) and Simulated Knockout with CellOracle

In order to better understanding and evaluating the knockout effect of key genes, simulated knockout and ATAC‐seq related base GRN was refined via CellOracle V1.2. First, the merge function of bedtools is used to merge all ATAC‐seq peaks (0, 24, and 48) with a maximum distance of 1000 bp between features allowed to be merged, which is used for containing as much as potential chromatin opening sites in early stage (i.e., within 48 h). Function ma.get_tss_info() of CellOracle is then used to annotate those merge peaks with hg38 human genome. Along with those annotated peaks, the base ATAC‐seq related Gene Regulation Network (GRN) was then refined using scRNA data of TF and DS samples. The cell‐type‐specific GRN's were trained using CellOracle's default procedure with balancedKNN implementation k = 1106, b_sight = 1106*8, b_maxl = 1106*4. To simulate the knockout effect of OLIG1, OLIG2, and OLIG3, the expression of those genes is set to 0. For the Markov chain simulation, parameters are set to steps = 200, duplication steps = 5.

### scRNA‐data Processing of dox‐inducible Samples

The processing of scRNA‐seq data of dox samples (sequenced by MGI) was similar to DS and TF samples. After the quality control process, 4728 cells and 50 059 genes were retained as 8 dox samples, and these samples were integrated with DS and TF samples by the same fastMMN function with default parameters.

### Calculation of the Proportion of Deep‐ and Upper‐layer Neurons

First, we identified neuron‐related cells based on the expression level of TUBB3 and MAP2. (Cells with expression level of TUBB3 or MAP2 greater than 2 were defined as neuron‐related cells.) Then, module scores^[^ ^134^ ^]^ for deep‐ layer markers (FEZF2,^[^ ^79^, ^80^ ^]^ BCL11B,^[^ ^79^, ^81^, ^82^ ^]^ SOX5,^[^ ^79^ ^]^ NFIA,^[^ ^83^ ^]^ NFIB,^[^ ^83^ ^]^ CELF1,^[^ ^84^ ^]^ TLE4,^[^ ^81^ ^]^ FOXP2,^[^ ^80^, ^82^ ^]^ TBR1^[^ ^84^ ^]^) and upper‐layer neurons markers (MEF2C,^[^ ^79^ ^]^ RORB,^[^ ^80^ ^]^ SATB2,^[^ ^79^, ^81^ ^]^ CUX1,^[^ ^49^, ^80^, ^85^ ^]^ CUX2,^[^ ^79^, ^85^ ^]^ POU3F2^[^ ^79^ ^]^) in each cell were calculated using AddModuleScore() function from Seurat package (V5.0.3). Based on these scores, neuron‐related cells with higher deep‐layer marker scores were finally defined as deep layer neurons, while the remainder were classified as upper‐layer neurons.

### CUT&RUN and ChIP‐seq

The hPSCs were transfected with Flag‐tagged 3N_TF mRNAs or induced for neurogenesis with dox‐inducible 3N_TF overexpression. During neuron induction with 3N_TF, cells were collected for CUT&RUN (cleavage under targets & release using nuclease) chromatin profiling using Hyperactive Universal CUT&Tag Assay Kit (vazyme, TD903). Antibody against Flag was used at 1:100 for 100 000 cells. DNA was collected for paired‐end sequencing.

The chromatin immunoprecipitation (ChIP) protocol for OLIG2 was adapted from published protocols.^[^ ^128^ ^]^ Briefly, cells were crosslinked with 1% formaldehyde for 10 min at room temperature and then treated with 0.2 M glycine to inactive the formaldehyde. Cells were then lysed to obtain chromatin extracts, which were sonicated to obtain DNA fragments with an average size of 300–500 bp. The resulting chromatin extracts were immunoprecipitated by OLIG2 antibody (Sigama, AB9610) immobilized on Protein‐G beads.

### ATAC‐seq

During neuron induction with dox‐inducible 3N_TF OE cell line, cells were collected at day0 (hPSCs), day1 (24hr) and day2 (48hr) for ATAC seq using Hyperactive ATAC‐Seq Library Prep Kit (vazyme, TD711). And then the DNA was submitted to GENEWIZ for paired‐end sequencing.

### Peak Info Processing

Software bowtie2^[^ ^135^ ^]^ and macs2^[^ ^136^ ^]^ is used to obtain the peak info of ChIP‐seq data as paired end sets. The Q value of ATAC‐seq callpeak is set to 0.01, and the Q value of ChIP‐seq callpeak is set to 0.05 (default parameters). Those peaks are annotated by annotatePeak function of R package ChIPseeker, and draw heatmaps by the plotHeatmap function of software deeptools^[^ ^137^ ^]^ with default parameters.

### Binding Site info Processing

To retain binding sites of target genes, we first merged all peaks from ChIP‐seq data with bedtools merge function by default parameters as well as duplicates within 0, 24, and 48 h of ATAC‐seq samples. Moreover, those merged ATAC‐seq peaks are combined with merged ChIP‐seq peaks by bedtools intersect function, which are annotated by R package TxDb.Hsapiens.UCSC.hg38.knownGene and ChIPseeker, and concerned as “binding sites” of each sample.

### Motif info Processing

The motif info of “binding sites” is processed by the software homer2.^[^ ^138^ ^]^ In brief, peak info of those “binding sites” are extracted from peak files within previous binding site info processing step, and then processed by function findMotifsGenome.pl with ‐size parameter 200 and ‐len parameter 12.

### Processing RNA‐seq Data (RNA‐seq Library Preparation)

During neuron induction with dox‐inducible 3N_TF OE cell line, cells were collected every 2hrs within 48hrs for RNA seq. Samples were collected in Trizol (Thermo Fisher Scientific, 15 596 018) and then submitted to GENEWIZ for paired‐end sequencing.

### Processing RNA‐seq Data (Peak info Processing)

To obtain the expression info of RNA‐seq, software hisat2,^[^ ^139^ ^]^ samtools, and stringtie are used to create gene count matrix from 0h to 48h. After the expression data matrix is obtained, count matrix was used to perform the differential gene analysis via the R software package DESeq2 3.17.^[^ ^140^ ^]^ The results of the differential analysis were screened as input data for the NMF analysis.

### Processing RNA‐seq Data (Non‐Negative Matrix Factorization (NMF) Analysis)

To cluster the time points from 0 to 48 h, the R package NMF^[^ ^141^ ^]^ 0.26 was employed for unsupervised clustering. The data were thresholded with differentially expressed genes based on padj < 0.05 and |log2FoldChange| > 0.5 in the CPM expression matrix. A preferred cluster result was chosen by considering the profiles of the cophenetic score and average silhouette width for clustering solutions ranging from 2 to 7 clusters. Based on the computed cophenetic results, the data was clustered into 5 clusters (C1‐C5), with the identification of C2 (0 h), C3 (2‐12 h), C4 (14‐26 h), C5 (28‐38 h), and C1 (40‐48 h) respectively.

### Processing RNA‐seq data (Weighted Gene Co‐Expression Network Analysis (WGCNA) Depend NMF)

Using the aforementioned CPM matrix and the clusters obtained from NMF clustering, WGCNA analysis was performed with the R package WGCNA 1.72.1 to identify modules most correlated with each cluster. Hub genes were obtained through module membership (MM), gene significance (GS), and manual filtering. Firstly, various β parameters were evaluated to construct a scale‐free network, with the power of β = 13 selected as the soft thresholding value. Secondly, the dynamic tree cut algorithm was applied with a minimal gene number of 30 within each module. Finally, trait‐related modules were selected based on the Pearson correlation coefficient between module eigengenes (ME) and sample characteristics of each module.

### Processing RNA‐seq Data (Construction of the Correlation Network)

For Figure S3f, initially, utilizing hub genes (KME, module eigengene‐based connectivity; |KME| > = 0.8) and the expression matrix as input data. For Figure 3e, utilizing hub genes together with ChIP identified binding peak TFs and ATAC enriched motif related TFs and the expression matrix as input data. The correlation matrix of hub genes was established through Pearson correlation analysis. Subsequently, the threshold for network connections was set at p‐value < 0.05 and |correlation| > 0.3. Finally, for Figure S3f (Supporting Information), data visualization was performed using the R package circlize 0.4.15. The result data were normalized using the logarithmic function, with the width of the lines in the graph representing the quantity of associations. For Figure 3e, data visualization was performed using Cytoscape 3.10.1,^[^ ^142^ ^]^ with the size of the nodes in the graph representing the quantity of associations, and the width of the lines representing the |correlation| between the two TFs.

### Processing RNA‐seq Data (Fuzzy C‐Means Clustering)

A fuzzy c‐means clustering^[^ ^143^, ^144^ ^]^ algorithm was utilized to generate gene clusters, allowing genes to belong to more than one cluster. The R package Mfuzz was employed for fuzzy c‐means cluster analysis. Initially, expression data underwent standardization, was transformed into the ExpressionSet format, missing values were removed, and data fuzziness was estimated. Finally, the mfuzz function was applied for the clustering process.

### GO‐term by DAVID

DAVID 6.8^[^ ^145^, ^146^ ^]^ (https://david.ncifcrf.gov/summary.jsp) was used for gene ontology analysis in Figures 3, 4, 6, 7; Figures S3h,j,S6i,S7h (Supporting Information).

### Statistical Analysis

Fiji ImageJ^[^ ^147^ ^]^ was used to analyze the image signal data. Statistical analyses were performed using GraphPad Prism 9 (GraphPad Software, San Diego, CA, USA). Raw data were normalized to control conditions where applicable (e.g., gene expression levels relative to housekeeping genes), and logarithmic transformation was applied for datasets with non‐normal distributions, as confirmed by Shapiro‐Wilk tests. Data are presented as mean ± standard deviation (SD) unless otherwise specified. Sample sizes (n) for each experiment are reported in the figure legends and ranged from n = 3–6 biological replicates. For comparisons between two groups, two‐sided Student's t‐test was used. For experiments with a single factor, one‐way analysis of variance (ANOVA) followed by Tukey's post hoc test was applied for multiple group comparisons. Significant differences are denoted as follows: *p < 0.05, **p < 0.01, ***p < 0.001, ****p < 0.0001.

## Conflict of Interest

The authors declare no conflict of interest.

## Author Contributions

L.Z. and W.W. contributed equally to this work. L.Z., N.X., and F.F. designed the study and wrote the paper with input from all authors. L.Z. conducted most of the experiments (including neuron induction, ATAC library preparation, OLIG KO cell line construction, TFs screening for facilitating neuronal maturation), analyzed the data, and prepared the figures. W.W. performed most of the bioinformatic analysis (including scRNA‐seq analysis, GRN analysis, ATAC‐seq and ChIP‐seq processing). J.Z. conducted electrophysiology‐related experiments and analyzed the data. L.L. conducted most of the scRNA library preparation, scRNA‐seq and ChIP‐seq library preparation. M.H. constructed most of the plasmids and synthesized mRNA used in this paper. L.D. synthesized mRNA used in this paper. S.F. and Q.Y. performed experimental work including ChIP and Western blot. B.P. and H.Q. conducted bioinformatic analysis, including NMF analysis, WGCNA analysis, and fuzzy clustering analysis. J.L. and R.A.R.P. provided essential intellectual input and designed some experiments.

## Supporting information



Supporting Information

Supporting Information

Supporting Video

## Data Availability

The data that support the findings of this study are available on request from the corresponding author. The data are not publicly available due to privacy or ethical restrictions.
