# Supplementary material for: Temporal Transcriptional Regulation of Human Neuronal Differentiation via Forward Programming
Source: Adv Sci (Weinh). 2025 Nov 23;13(6):e10641. doi: 10.1002/advs.202510641 (PMC12866710; doi:10.1002/advs.202510641)
Supplement: Supplementary file 2 — Supporting Information [file ADVS-13-e10641-s002.docx]

| **Primers for PCR** | **Sequence 5’-3’** |
| --- | --- |
| hMyt1l-Forward | CCTGCTCCTAAACGAAAGCCA |
| hMyt1l-Reverse | TCCCATCACTGTCGTCACACT |
| hCamk2a-Forward1 | ATTGAAGCCATAAGCAATGGAGA |
| hCamk2a-Reverse1 | TTCCGGGACCACAGGTTTTC |
| hCamk2a-Forward2 | GCTCTTCGAGGAATTGGGCAA |
| hCamk2a-Reverse2 | CCTCTGAGATGCTGTCATGTAGT |
| SYN1-Forward | TGGGGACTACTCCTCGTCAG |
| SYN1-Reverse | GTGGCCAGAAAGTCCAGCAT |
| NEFH Primer -Forward | CTGAGGAACACCAAGTGGGAGA |
| NEFH Primer -Reverse | TCCGACACTCTTCACCTTCCAG |
| STX1A Primer Forward | TGGAGAACAGCATCCGTGAGCT |
| STX1A Primer Reverse | CCTCTCCACATAGTCTACCGCG |
| DLG4 Forward | TCCACTCTGACAGTGAGACCGA |
| DLG4 Reverse | CGTCACTGTCTCGTAGCTCAGA |
| RIM Forward | CTCAGCCATCACCTTTCATGCC |
| RIM Reverse | CTGGAGGAACTACGCCAATACC |
| GAPDH-Forward | ACACCATGGGGAAGGTGAAG |
| GAPDH-Reverse | GTGACCAGGCGCCCAATA |
| HSP90AB1-Forward | CCTCACTAATGACTGGGAAGAC |
| HSP90AB1-Reverse | GGAGCCCGACGAGGAATAAA |
| OLIG1-RT-F | TTCCTCCTCCTCCACGACGG |
| OLIG1-RT-R | CTCGCGGCTGTTGATCTTGC |
| OLIG2-RT-F | AAATGACAGAGCCGGAGCTGC |
| OLIG2-RT-R | ACCAGTCGCTTCATCTCCTCCA |
| OLIG3-RT-F | GCGGGAGAGAGCAGCAAGTAC |
| OLIG3-RT-R | AGGTTCAGGTCGTGCATCCG |
| HSP-RT-F | CCTCACTAATGACTGGGAAGAC |
| HSP-RT-R | GGAGCCCGACGAGGAATAAA |
| OLIG2-cut-F | GGACAAGCTAGGAGGCAGTGG |
| OLIG2-cut-R | GCAGACGGAGACTTGAGTAGGC |
| OLIG3-cut-R | GCGCCTTGAGTAGCGAGTG |
| OLIG3-cut-F | GCGGGAGAGAGCAGCAAGTAC |

**Table S1. Primers for PCR**

**Table S2. Cell type marker genes**

| Cell Type | Gene Symbol | Reference |
| --- | --- | --- |
| Neuroepithelial cells | CDH1, SOX2, ZO-1 | PMID: 22276221;  PMID: 37192616;  PMID: 33723434. |
| Radial glial | FABP7, CDH2, GFAP, NES, VIM, SOX2, PAX6, HES1, HES5, SOX9 | PMID: 33723434;  PMID: 37192616;  PMID: 36466166;  PMID: 38194967. |
| Intermediate progenitor cells | EOMES | PMID: 37192616. |
| Neuroblast | DCX, TUBB3, NACM1 | PMID: 34798047;  PMID: 31067457. |
| Mature Neurons | MAP2, NEFM, GAP43, ENO2 | PMID: 33470930;  PMID: 2720952;  PMID: 39429212. |

**Table S3. Key Resource Table**

***LIFE SCIENCES***

| **REAGENT or RESOURCE** | **SOURCE** | **IDENTIFIER** |
| --- | --- | --- |
| Antibodies | | |
| Rabbit monoclonal anti-SATB2 | ABclonal | Cat# A19837;  RRID: AB_3083559 |
| Rabbit polyclonal anti-MAP2 | proteintech | Cat# 17490-1-AP;  RRID: AB_2137880 |
| Rabbit polyclonal anti-VGLUT2 | Affinity | Cat# DF13296;  RRID: AB_2846315 |
| Rabbit polyclonal anti-TBR1 | proteintech | Cat# 20932-1-AP;  RRID: AB_10695502 |
| Mouse monoclonal anti-TUBB3 | BioLegend | Cat# 801201;  RRID: AB_2313773 |
| Rabbit monoclonal anti-OLIG3 | Abcam | Cat# ab129197;  RRID: AB_11142825 |
| Rabbit polyclonal anti-OLIG2 | Sigma-Aldrich | Cat# AB9610,  RRID: AB_570666 |
| Mouse monoclonal anti-FLAG | Sigma-Aldrich | Cat# F1804;  RRID: AB_262044 |
| Mouse monoclonal anti-DLG4 | neuromab | Cat# 75-028;  RRID: AB_2292909 |
| Rabbit polyclonal anti-RIM | Synaptic Systems | Cat# 140 213;  RRID: AB_2832237 |
| Rabbit monoclonal anti-CDH1 | CST | Cat# 3195;  RRID: AB_2291471 |
| Rabbit monoclonal anti-CDH2 | CST | Cat# 13116;  RRID: AB_2687616 |
| Rabbit monoclonal anti-EOMES | CST | Cat# 81493;  RRID: AB_2799974 |
| Anti-rabbbit IgG, HRP-linked antibody | CST | Cat# 7074;  RRID: AB_2099233 |
| Alexa Fluor 594-A ffiniPure Donkey anti-Rabbit IgG(H+L) | Jackson ImmunoResearch Labs | Cat# 711-585-152,  RRID: AB_2340621 |
| Alexa Fluor 594-A ffiniPure Donkey anti-Mouse IgG(H+L) | Jackson ImmunoResearch Labs | Cat# 715-585-150,  RRID: AB_2340854 |
| Alexa Fluor 488-A ffiniPure Donkey anti-Rabbit IgG(H+L) | Jackson ImmunoResearch Labs | Cat# 711-545-152,  RRID: AB_2313584 |
| Alexa Fluor 488-A ffiniPure Donkey anti-Mouse IgG(H+L) | Jackson ImmunoResearch Labs | Cat# 715-545-150,  RRID: AB_2340846 |
| Bacterial and virus strains | | |
| pCR2-UTR-R1R2 | KO paper | N/A |
| PCR2-PUC57-NEUROG1-FLAG | This paper | N/A |
| PCR2-PUC57-NEUROG2-FLAG | This paper | N/A |
| PCR2-PUC57-NEUROD1-FLAG | This paper | N/A |
| PCR2-PUC57-NEUROG1 | This paper | N/A |
| PCR2-PUC57-NEUROG2 | This paper | N/A |
| PCR2-PUC57-NEUROD1 | This paper | N/A |
| TFORF-NEUROD2 | This paper | N/A |
| TFORF-MSC | This paper | N/A |
| TFORF-TLE1 | This paper | N/A |
| TFORF-PAX3 | This paper | N/A |
| TFORF-JUNB | This paper | N/A |
| CLYBL-TO-hNEURO_TFs-BSD-mApple | This paper | N/A |
| pLV-eGFP | This paper | N/A |
| CLYBL-TO-hNGN2-BSD-mApple | PMID: 31422865 | Addgene Cat#124229 |
| Chemicals, peptides, and recombinant proteins | | |
| SB431542 | TargetMol | T1726;  CAS 301836-41-9 |
| LDN193189 | TargetMol | T1935;  CAS 1062368-24-4 |
| Y27632 | TargetMol | T1725;  CAS 129830-38-2 |
| SU5402 | TargetMol | T6996;  CAS 215543-92-3 |
| DAPT | TargetMol | T6202;  CAS 208255-80-5 |
| XAV939 | TargetMol | T1878  CAS 284028-89-3 |
| PD0325901 | TargetMol | T6189;  CAS 391210-10-9 |
| cAMP | Sigma | D0627 |
| BDNF | CELLAPY | CA32006 |
| N2 | Gibco | 17502048 |
| B27 | Gibco | 12587010 |
| Matrigel | Corning | 354277 |
| Bovine serum albumin | Sangon Biotech | A600903-0010 |
| SYBR Green | Vazyme | Q111-02 |
| RT-PCR Master Mix | Vazyme | R323 |
| TritonX-100 | Biosharp | BS084 |
| mTeSR1 medium | STEMCELL Technologies | 85850 |
| Neural induction medium | STEMCELL Technologies | 05835 |
| Accutase | Sigma | A6964 |
| Donkey serum | Jackson ImmunoResearch | RRID: AB_2337258 |
| Neurobasal medium | Gibco | 21103049 |
| Doxycycline hyclate | TargetMol | T1687L |
| Trizol | Thermo Fisher Scientific | 15596018 |
| Critical commercial assays | | |
| Hyperactive® Universal CUT&Tag Assay Kit for Illumina | vazyme | TD903 |
| Hyperactive® Universal CUT&Tag Assay Kit for Illumina | vazyme | TD904 |
| Hyperactive ATAC-Seq Library Prep Kit for Illumina | vazyme | TD711 |
| DNBelab C Series Single-Cell ATAC Library Prep set | MGI | 1000021878 |
| mRNA synthesis kit | Thermo Fisher Scientific, MEGAscript T7 kit (Ambion, Austin, TX) | AMB13345 |
| Deposited data | | |
| Single cell RNA seq (3N_TF mRNA overexpression in hPSCs female cell line) | This paper | Accession number: GSE286357 |
| Single cell RNA seq (dox-inducible 3N_TF overexpression in hPSCs female cell line) | This paper | Accession number: GSE286357 |
| Single cell RNA seq (dox-inducible 3N_TF overexpression in hPSCs male cell line) | This paper | Accession number: GSE286357 |
| Bulk RNA seq | This paper | Accession number: GSE285570 |
| ATAC seq | This paper | Accession number: GSE285531 |
| CUT&Tag (NEUROG1, NEUROG2, NEUROD1, NEUROD2, MSC, OLIG2) | This paper | Accession number: GSE285532 |
| Human developing brain data | PMID: 37192616 | GSE155121 |
| Bulk RNA seq (NEUROG2 overexpression in hPSCs) | PMID: 38446849 | Accession number: GSE181019 |
| Experimental models: Cell lines | | |
| Human: Passage 40 hPSCs cells (female) | Cellapy company | N/A |
| Human: Passage 30 hPSCs cells (male) | Cellapy company | N/A |
| Dox inducible 3N_TF OE hPSCs | This paper | N/A |
| OLIG1 KO in Dox inducible 3N_TF OE hPSCs | This paper | N/A |
| OLIG2 KO in Dox inducible 3N_TF OE hPSCs | This paper | N/A |
| OLIG3 KO in Dox inducible 3N_TF OE hPSCs | This paper | N/A |
| OLIG KO in Dox inducible 3N_TF OE hPSCs | This paper | N/A |
| Oligonucleotides | | |
| Primers for RT-PCR, see Table 1 | This paper | N/A |
| Recombinant DNA | | |
| pCR2-UTR-R1R2 | PMID: 28205555 | N/A |
| PCR2-PUC57-NEUROG1-FLAG | This paper | N/A |
| PCR2-PUC57-NEUROG2-FLAG | This paper | N/A |
| PCR2-PUC57-NEUROD1-FLAG | This paper | N/A |
| PCR2-PUC57-NEUROG1 | This paper | N/A |
| PCR2-PUC57-NEUROG2 | This paper | N/A |
| PCR2-PUC57-NEUROD1 | This paper | N/A |
| pLV-eGFP | This paper | N/A |
| TFORF-NEUROD2 | This paper | N/A |
| TFORF-MSC | This paper | N/A |
| TFORF-TLE1 | This paper | N/A |
| TFORF-PAX3 | This paper | N/A |
| TFORF-JUNB | This paper | N/A |
| PSIN-flag-zsGreen | This paper | N/A |
| PSIN-flag-NEUROD2 | This paper | N/A |
| PSIN-flag-MSC | This paper | N/A |
| Software and algorithms | | |
| ImageJ | PMID: 22743772 | https://imagej.nih.gov/ij/ |
| Bowtie2 | PMID: 22388286 | http://bowtie-bio.sourceforge.net/bowtie2/index.shtml |
| Macs2 | PMID: 18798982 |  |
| DNBelab C Series scRNA analysis software |  | https://github.com/MGI-tech-bioinformatics/DNBelab_C_Series_scRNA-analysis-software |
| DNBelab C Series HT scRNA analysis software |  | https://github.com/MGI-tech-bioinformatics/DNBelab_C_Series_HT_scRNA-analysis-software |
| scvelo V0.3.1 | PMID: 32747759 |  |
| deeptools | PMID: 27079975 |  |
| Homer2 | PMID: 26157991 |  |
| Hisat2 | PMID: 34103331 |  |
| DESeq2 3.17 | PMID: 25516281 |  |
| Monocle3 algorithm | PMID: 24658644 |  |
| non-negative matrix factorization algorithm |  | <https://doi.org/10.48550/arXiv.cs/0408058> |
| fuzzy c-means clustering algorithm | PMID: 16078370  PMID: 18084642 |  |
