# Supplementary material for: Temporal Transcriptional Regulation of Human Neuronal Differentiation via Forward Programming
Source: Adv Sci (Weinh). 2025 Nov 23;13(6):e10641. doi: 10.1002/advs.202510641 (PMC12866710; doi:10.1002/advs.202510641)
Supplement: Supplementary file 1 — Supporting Information [file ADVS-13-e10641-s003.docx]

**Figure S1. Identification of an mRNA-based recipe for rapid neurogenesis using established 3N_TF and characterization of induced neurons.**

**a,** Neuronal differentiation induced by different combination of TFs encoded by mRNAs was shown. Expression of TUBB3 and DAPI was measured at day 7 post-transfection by immunocytochemistry. Two hPSCs cell lines (hPSCs-female and hPSCs-male) were used. Scale bars indicate 25μm.

**b,** Percentage of cells with positive TUBB3 staining in the culture was counted for each condition. Data represent the mean ± SD of n = 3 independent replicates.

**c,** A screen for optimal culture media for 3N_TF induced neurogenesis. Expression of TUBB3, MAP2, and DAPI was measured at day 7 post-transfection by immunocytochemistry. Two hPSCs cell lines (hPSCs-female and hPSCs-male) were used. Scale bars indicate 25μm**.**

**d,** Percentage of cells with positive MAP2 and TUBB3 staining in the culture was counted for each culture media. Data represent the mean ± SD of n = 3 independent replicates.

**e,** Representative immunofluorescence images for TUBB3 and MAP2 in 3N_TF induced neurons. hPSCs-female cell line was used. Scale bars are 100μm.

**f,** Heatmap depiction of time course expression of neuronal marker genes. Cells were harvested at day0, day5, day7 and day9 post-induction of 3N_TF for RNA-seq analysis. hPSCs-female cell line was used.

**g,** Electrophysiological properties of 3N_TF induced neurons. Representative traces of whole-cell voltage-clamp Na+ and K+ currents at day15. hPSCs-female cell line was used.

**h,** Quantification of current/voltage (I/V) curves of Na+ currents. Data represent the mean ± SD of n = 3 independent replicates.

**i,** Quantification of current/voltage (I/V) curves of K+ currents. Cells were step-depolarized, fast-activating and inactivating inward Na+ currents as well as outward K+ currents were observed, indicating that the induced neurons had active membrane properties. Data represent the mean ± SD of n = 3 independent replicates.

**j,** Representative traces of spontaneous action potentials recorded from induced neurons at day34. The data indicates that the induced neurons exhibited prominent after-hyperpolarization potentials following action potentials (APs).

**k,** Membrane potential after induction over time, the resting membrane potential of neuronal cells was around -40 mv.

**l,** Representative traces of APs in response to step current injection 34 days after induction. This result suggests that the induced neurons exhibited prominent after-hyperpolarization potentials following APs.

**m,** The number of APs generated by step-current injection 34 days after induction.

**Figure S2. Transcriptomic and cellular analysis of TF- and DS-induced neuronal differentiation.**

**a,** Violin plots showing the expression of *PAX6*, *SOX1, NR2F1, OLIG1, OLIG2 and OLIG3.*

**b,** The correlation score was calculated between *in vitro* differentiated neural endpoint cells and developing brain samples (PCW3-PCW12 stage, GSE155121) based on their transcriptomic profiles. Cells differentiated via 3N_TF and DS protocols were colored according to the correlation score, while reference brain samples (GSE155121) were colored grey. Distribution of cell-type annotations across different Pearson correlation ranges. Stacked bar plots show the proportion of reference-annotated cell types within each Pearson correlation range. Higher correlation ranges (0.6-0.8) are predominantly composed of neuronal cell types such as glutamatergic neurons.

**c,** Dot plots showing marker genes expression patterns. The size of dot represents the percentage of cells expressing the corresponding gene and the color intensity of dot represents scaled average expression of the gene.

**d,** Schematic of neuronal differentiation in doxycycline-inducible system. Time points at which cells were harvested for scRNA-seq profiling are marked with red circles. Two hPSCs cell lines (hPSCs-female and hPSCs-male) were used. dox, doxycycline. Scale bars are 210μm.

**e,** UMAP plot of cells from mRNA-based forward programming (TF), Dual-SMAD inhibition induced differentiation (DS), and dox-inducible forward programming. Cells are colored according to their differentiation methods and the differentiation date. hPSCs-female cell line was used.

**f,** UMAP embeddings of scRNA-seq data from the differentiation of a male hPSC line into female hPSC data, with cells colored by their source.

**g,** The correlation score between scRNA-seq data from the male hPSC line and the female hPSC line.

**h,** UMAPs depicting the expression of *CDH1* and *CDH2* expression. The color scale bar indicates the level of gene expression.

**i,** Representative immunofluorescence images for CDH1 and CDH2 in 3N_TF induced neurons and DS inhibition induced neurons. Scale bars are 10μm. hPSCs-female cell line was used.

**j,** UMAPs depicting the expression of *EOMES* expression. The color scale bar indicates the level of gene expression.

**k,** Representative immunofluorescence images for EOMES in 3N_TF induced neurons and DS inhibition induced neurons. Scale bars are 10μm. hPSCs-female cell line was used.

**Figure S3. Time-course multi-omics analyses reveal gene regulatory cascades.**

**a,** A venn diagram showing the overlaps of NEUROG1, NEUROG2, and NEUROD1 ChIP-seq binding peaks. hPSCs-female cell line was used for this analysis.

**b,** Bar charts showing the percentages of NEUROG1, NEUROG2, and NEUROD1 binding peaks distribution.

**c,** Bar charts showing the percentages of ATAC peaks distribution in the genome. hPSCs-female cell line was used for this analysis.

**d,** Heat map of non-negative matrix factorization (NMF) cophenetic correlation coefficient and consensus matrix. On the basis of visual inspection of the hierarchical clustering and the profiles of cophenetic correlation coefficient for solutions with 2 to 7 clusters, we consider Rank = 5 to be the preferred solution (indicated by black triangles) and use this solution to arrange the samples shown in Fig3B and 3C (yielding the five clusters labeled as “0hr”, “2-12hr”, “14-26hr”, “28-38hr”, “40-48hr”). hPSCs-female cell line was used for this analysis.

**e**, NMF consensus-clustering identified five distinct molecular states (0hr, 2-12hr, 14-26hr, 28-38hr, and 40-48hr) within 48hrs of differentiation. NMF, non-negative matrix factorization.

**f**, Chords representation of the correlation of TFs in the five selected modules (|KME|>=0.8) and 3N_TF. The width of the lines in the graph representing the quantity of associations between the two modules.

**g,** During neuron differentiation (0-12hr), cells extended filopodia and lamellipodia-like veils (indicated by red triangles) and migrated. Meanwhile, growth cones (indicated by white triangles) facilitated axon growth. hPSCs-female cell line was used for this analysis.

**h,** GO analysis of TFs (TF |KME|>=0.8) in the co-expression modules highlighted with black boxes in Figure 3b. Enriched GO terms are shown vertically on the right, and the negative logarithmic p-values are shown by the bar graphs below. KME, module eigengene-based connectivity.

**i,** Fuzzy clustering analysis of genes showing distinct expression patterns within the first 48 hrs of neuronal induction.

**j,** GO analysis of genes identified by fuzzy clustering. The bar graphs show the negative logarithmic p-values.

**k,** Genome browser visualization of the chromatin accessibility and 3N_TF binding tracks within specific gene loci (chromatin remodeling and organization related genes: *CTCF, SMARCB1, SMARCA4*; neuron related genes: *ATOH8, OLIG2, EBF2, ZEB2*; notch signaling pathway related genes: *DLL3, HES6*) at different neuron induction time points (hPSCs, day1 and day2).

**Figure S4. Analysis of the heterogeneity of endpoint cells induced by 3N_TF and DS inhibition.**

**a,** UMAP shown the clustering results of clusters 5 and 6 of Figure 2a, representing endpoint cells induced by 3N_TF and DS inhibition.

**b,** Dot plots showing marker genes expression patterns for each cluster. The size of dot represents the percentage of cells expressing the corresponding gene and the color intensity of dot represents scaled average expression of the gene in a given cluster.

**c,** UMAP visualization showing the annotated cell types (Glutamatergic, GABAergic and PRPH^+^ neurons).

**d,** UMAP projections illustrating the mapping of annotated cell types (Glutamatergic, GABAergic and PRPH^+^ neurons) onto the UMAP space depicted in Figure 2a.

**e,** Time-dependent analysis of cell cluster proportions during neuronal differentiation progression of 3N_TF and DS inhibition.

**f,** Stacked bar plot showing the proportions of cell types identified through scRNA-seq. hPSCs, induced pluripotent stem cells; NE, neuroepithelial; RG, radial glia; NB, neuroblast; IP, intermediate progenitor; DRG, dividing radial glia; NC, neural crest.

**g,** Dot plot displaying marker gene expression patterns in specific samples. The size of dot represents the relative gene expression percentage for each sample, while the color intensity indicates the average expression of the gene in that sample.

**h and i,** UMAP of different cell lines at differentiation day 15 cells. The clusters were annotated based on the expression of marker genes.

**j,** UMAPs depicting the expression of GABA (*GAD1, GAD2, SLC32A1*), glutamatergic (*SLC17A6, SLC17A7, GLS*) and peripheral (*PRPH*) related genes.

**k**, UMAPs depicting the expression of *EBF1 and EBF2* expression. The color scale bar indicates the level of gene expression.

 **Figure S5. UMAP embeddings of the mean scRNA expression for subnetwork-specific genes.**

Color bars on the left of each UMAP indicate mean expression of subnetwork-specific genes. Top genes of related subnetwork ranked by out-degree value are shown vertically on the right to the UMAP of each subnetwork. The length of bars represents the out-degree values. Out-degree values indicate the number of outward directed graph edges (filtered correlation between genes) from the given graph vertex (which mean regulators).

**Figure S6. Knockout of OLIG genes results in a delay in neurogenesis.**

**a**, Cell density plot of development flow and *in silico* *OLIG* KO simulation by CellOracle in the force-directed graph embedding space.

**b,** Schematic images and sequence results of dual sgRNA-mediated *OLIG1, OLIG2,* and *OLIG3* knockout in hPSCs using CRISRP/Cas9 technology.

**c,** Verification of successful gene knockout by PCR analysis. The results for wild type and knockout strains are shown using knockout specific primer pairs (Supplementary table1). hPSCs-female cell line was used for this analysis.

**d,** Verification of successful OLIG2 knockout by western blot analysis. Representative western blot analysis of the expression level of OLIG2 protein at day3 in induced neurons derived from WT and *OLIG* KO cell lines. hPSCs-female cell line was used for this analysis.

**e,** Verification of successful OLIG2 and OLIG3 knockout by immunofluorescence analysis. Representative immunofluorescence images for OLIG2 and OLIG3 at day4 of induced neurons from WT and *OLIG* KO cell line. Scale bars are 40μm.

**f,** Schematic of neuron induction and scRNA-seq analysis using *OLIG* KO cell line. Time points at which cells were harvested for scRNA-seq profiling are marked with red circles.

**g,** UMAP visualization of WT and *OLIG* KO differentiated cells. Cells are divided into 23 clusters indicated by different colors.

**h,** UMAP embeddings of mean scRNA expression of subnetwork-specific genes showing three highly cell-type-specific subnetworks. Subnetwork-specific genes associated with WT route are colored blue, those associated with the *OLIG* KO route are colored red, and specific genes shared between WT and *OLIG* KO route are colored green.

**i**, Gene ontology enrichment analysis by DAVID of top 200 ranked genes in subnetwork associated with WT, OLIG or shared between WT and *OLIG* KO route, and the bar graph shows the negative logarithmic p-values.

**j,** GENIE3 GRN analysis illustrating the top regulators clustered into subnetworks. Regulators were selected based on their predicted regulation of the top differentially expressed genes in clusters associated with WT and *OLIG* KO route. Genes labeled within each subnetwork are associated with neuron development (*NEUROD1, NEUROD2, NR2F6, XBP1, OLIG1, OLIG2*), cell proliferation (*CDK1, CDK2, EZH2*) and the NOTCH signaling pathway (*HES1*).

**Figure S7****. Overexpression of NEUROD2 and MSC expedites neuronal maturation in both TF- and DS-induced differentiation system.**

**a**, UMAPs depicting the expression of neuronal maturation related genes. The color scale bar indicates the level of gene expression.

**b,** In conjunction with 3N_TF, MSC were overexpressed using mRNA, and the expression levels of genes related neuronal maturation were examined at day15. Data represent the mean ± SD of n = 3 independent replicates. hPSCs-female cell line was used for this analysis. *p < 0.05; **p < 0.01; ***p < 0.001; ****p < 0.0001.

**c**, Representative immunofluorescence images for DLG4 and RIM in 3N_TF induced neurons at day30. hPSCs-female cell line was used for this analysis. Scale bars are 10μm.

**d**, Quantification of the fluorescence intensity of DLG4 and RIM staining. Data are presented as mean ± SD of n = 3 independent replicates. *p < 0.05; **p < 0.01; ***p < 0.001; ****p < 0.0001.

**e**, Representative voltage clamp recordings of spontaneous excitatory postsynaptic currents (sEPSCs) from 3N_TF induced neurons at day 25 for each condition. hPSCs-female cell line was used for this analysis.

**f**, The quantification of the frequency and amplitude of sEPSCs. Data represent the mean ± SD of n = 3 independent replicates. *p < 0.05; **p < 0.01; ***p < 0.001; ****p < 0.0001.

**g,** Heatmap illustrating the expression of genes related to neuronal excitability and connectivity at day11.

**h**, Enriched gene ontology terms for upregulated genes that were bound by MSC.

**i**, ChIP-seq profile of flag-MSC within ±3 kb from the peak center (red signal) in neurons induced with 3N_TF and flag-MSC at day10. A total of 45 peaks were identified using the macs2 with default parameters.

**j**, Genome browser visualization of the MSC binding tracks within specific gene loci.

**k**, Using DS protocol, NEUROD2 were overexpressed using lentivirus, and the expression levels of genes related neuronal maturation were examined at differentiation at day 20. Data represent the mean ± SD of n = 3 independent replicates. hPSCs-male cell line was used for this analysis. *p < 0.05; **p < 0.01; ***p < 0.001; ****p < 0.0001.

**l**, Representative immunofluorescence images for DLG4 in DS induced neurons at day30. hPSCs-male cell line was used for this analysis. Scale bars are 100μm.

**m**, Quantification of the fluorescence intensity of DLG4 staining. Data are presented as mean ± SD of n = 3 independent replicates. *p < 0.05; **p < 0.01; ***p < 0.001; ****p < 0.0001.

**n**, Using DS protocol, MSC were overexpressed using lentivirus, and the expression levels of genes related neuronal maturation were examined at differentiation at day 20. Data are presented as mean ± SD of n = 3 independent replicates. hPSCs-male cell line was used for this analysis. *p < 0.05; **p < 0.01; ***p < 0.001; ****p < 0.0001.

**o**, Representative immunofluorescence images for DLG4 in DS induced neurons at day30. hPSCs-male cell line was used for this analysis. Scale bars are 100μm.

**p**, Quantification of the fluorescence intensity of DLG4 staining. Data are presented as mean ± SD of n = 3 independent replicates. *p < 0.05; **p < 0.01; ***p < 0.001; ****p < 0.0001.

**Video 1. Tight-junction hPSCs rapidly dispersed following TFs-induced reprogramming.**

After overexpression of 3N_TF, live cells were imaged for over about 50 hours. The video shows that tight-junction hPSCs rapidly dispersed within 24 hours and then form thin and elongated cells toward neurogenesis within 50 hours.
